# Dissociative Electron Attachment in 1,3-Dimethyl-5-Thiocyanatouracil at Different Hydration Conditions

**DOI:** 10.1021/acs.jpcb.6c05644

**Published:** 2026-10-26

**Authors:** Adrian Szczyrba, Samanta Makurat, Vy T. T. Nguyen, Jiakuan Chen, Magdalena Datta, Karol Biernacki, Jaroslav Kočišek, Jozef Ďurana, Barbora Sedmidubská, Sebastian Demkowicz, Stephan Denifl, Janusz Rak

**Affiliations:** † Laboratory of Biological Sensitizers, Physical Chemistry Department, Faculty of Chemistry, 49646 University of Gdańsk, Gdańsk 80-308, Poland; ‡ Institut für Ionenphysik und Angewandte Physik and Center for Biomolecular Sciences Innsbruck, 27255 Universität Innsbruck, Technikerstrasse 25, Innsbruck A-6020, Austria; § Department of Inorganic Chemistry, Faculty of Chemistry, Gdańsk University of Technology, Narutowicza 11/12, Gdańsk 80-233, Poland; ∥ Department of Organic Chemistry, Faculty of Chemistry, 49557 Gdańsk University of Technology, Narutowicza 11/12, Gdańsk 80-233, Poland; ⊥ Heyrovský Institute of the Czech Academy of Sciences, v.v.i., Dolejškova 3, Prague 18200, Czech Republic; # University of Chemistry and Technology Prague, Technická 5, Prague 6 166 28, Czech Republic; ∇ Institute of Physics of the Czech Academy of Sciences, v.v.i., Na Slovance 1999/2, Prague 8 182 00, Czech Republic

## Abstract

5-Thiocyanato-2′-deoxyuridine has emerged as a promising radiosensitizer for cancer radiotherapy. Upon X-ray irradiation, hydrated electrons generated in the tumor microenvironment can interact with this molecule, forming reactive transient species capable of inducing damage to DNA, the primary target of radiotherapy. In this work, we investigate dissociative electron attachment (DEA) to 1,3-dimethyl-5-thiocyanatouracil, a doubly methylated analogue of 5-thiocyanato-2′-deoxyuridine, in both the gas phase and microhydrated environments. Gas-phase measurements were performed using a crossed electron–molecular beam apparatus coupled with mass spectrometry, while microhydrated clusters were examined using a CLUster-Beam setup, enabling direct assessment of hydration effects on DEA. The experimental results are supported by quantum chemical calculations, providing insight into thermodynamic and kinetic parameters, including electron affinities, reaction thresholds, and activation barriers. In both environments, the parent anion and the CN^–^ fragment dominate the product distribution, accompanied by minor channels such as SCN^–^ formation. Microhydration leads to a moderate suppression of the S–CN bond cleavage pathway but does not inhibit the reaction. Overall, these findings demonstrate that microhydration modulates, yet preserves, DEA activity in this class of radiosensitizers, supporting their potential effectiveness under biologically relevant conditions.

## Graphical abstract

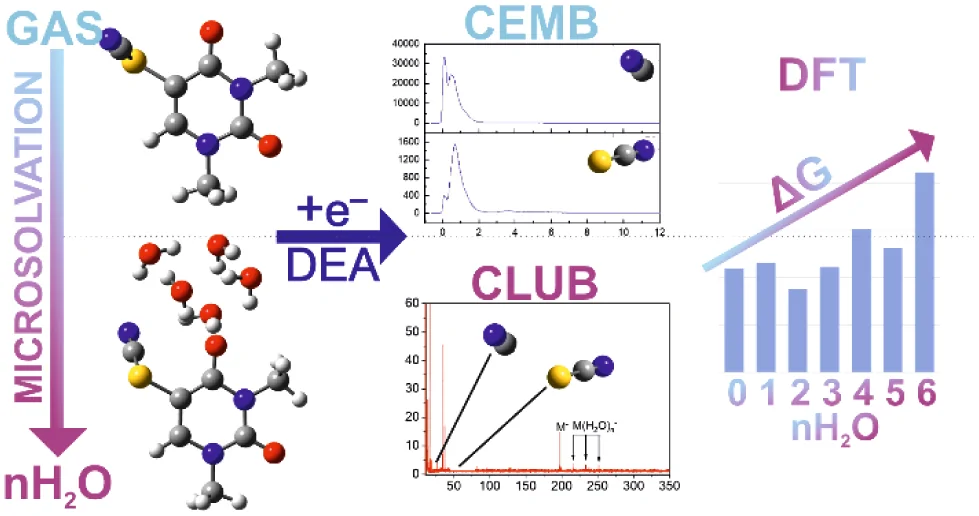

## 1 Introduction

Radiation therapy, one of the principal modalities for the treatment of solid tumors,^1^ is limited by the oxygen effect.^2^ Specifically, molecular oxygen dissolved in the cellular environment is required to “fix” DNA damage^3,4^ induced by reactive species generated during water radiolysis. Because many solid tumors are characterized by hypoxic conditions,^5^ the efficacy of ionizing radiation is consequently reduced.^6,7^ Small molecules acting as radiosensitizers can mitigate this limitation by restoring the radiation sensitivity of hypoxic cells.^8,9^

To date, several classes of radiosensitizers have been reported, including oxygen mimetics,^9^ modified nucleosides,^10^ metal nanoparticles, and metal complexes.^11^ Among modified nucleosides, 5-bromo-2′-deoxyuridine (BrdU) is considered a prototypical radiosensitizer. Its radiosensitizing properties were first investigated by the Szybalski group in the 1960s,^12^ further explored in the 1980s-1990s,^13,14^ and, since the early 2000s, studied extensively by our group^10,15,16^ and others.^17–19^

In nonsensitized cells, hydroxyl radicals are regarded as the primary agents responsible for radiation-induced DNA damage.^20^ In contrast, in the presence of BrdU and related analogues, solvated electrons—produced during water radiolysis in yields comparable to hydroxyl radicals^21,22^ —play a crucial role. During DNA replication, BrdU is incorporated into the DNA of proliferating cells.^23–25^ Subsequent capture of a solvated electron by BrdU leads to prompt DEA, generating a highly reactive radical species.^16^ This radical undergoes further transformation, ultimately inducing a single-strand break (SSB) in DNA.^26,27^ Accumulation of a sufficient number of such lesions triggers apoptotic pathways, resulting in the selective elimination of BrdU-labeled cells.^28^

The vulnerability to DEA for various uracil derivatives, was studied at the quantum chemical level in a water solution modeled with PCM (Polarized Continuum Model) by Chomicz and coworkers.^10,29^ They demonstrated the efficient formation of reactive uracil-5-yl radicals upon electron attachment. Izadi and Saqib by using Crossed Electron-Molecular Beam (CEMB) experiments coupled with theoretical modeling explained the DEA processes occurring upon electron attachment in the case of 5-bromo-4-thio-2′-deoxyuridine (BrSdU), 5-bromo-4-thiouracil (BrSU), and 5-iodo-4-thiouracil (ISdU), showing how the halogen substituent influences the DEA.^30,31^ In the other investigation of electron attachment to the metronidazole (the potential radiosensitizer in the group of oxygen mimetics) conducted by Lochmann et al., they found that microhydratation plays a crucial role in the DEA process by suppressing some of the dissociation pathways.^32^

In biological environments, radiosensitizing nucleosides can be activated through the attachment of solvated electrons, generating reactive transient species capable of inducing DNA damage.^33^ For a compound to act as a radiosensitizer via this mechanism, the presence of water should not suppress DEA pathways leading to such damaging species.^34^ Accordingly, a propensity for DEA both in the gas phase and under microsolvated conditions is a key requirement for nucleobase–based radiosensitizers. This distinction is illustrated by 5-cyanouracil, which, despite a relatively large gas-phase adiabatic electron affinity (AEA) of approximately 3 eV and the formation of stable parent anions observed in anion photoelectron spectra, does not undergo DEA.^10^ Consequently, it lacks radiosensitizing activity.

In this work, we investigate the interaction of low-energy electrons with 1,3-dimethyl-5-thiocyanatouracil (SCNdMeU) in both isolated and microhydrated environments to elucidate the role of hydration in the radiosensitization process. One hypothesis suggests that microsolvation may suppress DEA, potentially diminishing radiosensitizer efficiency. To address this, we combine crossed electron–molecular beam (CEMB) and CLUster Beam (CLUB) experiments with molecular dynamics and quantum chemical calculations. Our results demonstrate that hydration increases the kinetic barrier for a key dissociation pathway, providing direct insight into how water modulates DEA-driven reactivity relevant to radiosensitization.

For clarity, it is important to distinguish between electron-induced reactivity and radiosensitizing activity. Our study unequivocally demonstrates the former for SCNdMeU in the gas phase and under microsolvated conditions. Radiosensitization, however, refers to an increase in cellular sensitivity to ionizing radiation. Although electron-induced reactivity is a prerequisite for the proposed radiosensitizing mechanism, favorable reactivity alone does not establish radiosensitizing efficacy since such efficacy also depends on factors including cellular uptake, resistance to enzymatic degradation, and the ability of radical products generated through dissociative electron attachment (DEA) to persist long enough to damage DNA. Nevertheless, it is worth emphasizing here that the radiosensitizing activity of a structurally related compound, 5-thiocyanato-2′-deoxyuridine, has already been demonstrated in PC3 cells.^35^

## 2 Methods

### 2.1 Synthesis of 1,3-Dimethyl-5-thiocyanatouracil

SCNdMeU was synthesized according to the method described by Noikham and Yotphan.^36^ Its purity, MS and ^1^HNMR spectra are gathered in Supporting Information (see Figures S1, S2 and S3).

### 2.2 Experimental Section

#### 2.2.1 CEMB Experiment

A crossed electron-molecular beam instrument was used for the electron attachment study with the isolated molecule in the gas phase. The basic components of the setup located at the University of Innsbruck are as follows,^37^ (i) a neutral beam source, (ii) an electron source, (iii) a quadrupole mass spectrometer and last, (iv) the ion detection system. A sample container with a glass inset, located inside the vacuum chamber, was connected to a 1 mm diameter capillary tube made of copper. The tip of the capillary was placed about 2 cm in front of the interaction region with the electron beam. The sample was used as received. The reported measurements for negative ions were made with the oven temperature of about 351 K and a working pressure of about 1 × 10^–7^ mbar in the vacuum chamber. The electron beam was generated by means of a hemispherical electron monochromator (HEM). The HEM consists of three elements: (i) a hairpin filament with a current of 2.35 A that emits and forms the electron beam, (ii) two concentric hemispherical surfaces, each with different electric potential, so an electric field is formed for selection of the kinetic energy of the electrons, and (iii) two stacks of electrostatic lenses. Those are used for focus and guidance of the electron beam from the filament into the collision chamber where electrons interact with the neutral molecules. The energy resolution of the beam was about 140 meV for full width at half-maximum (fwhm) which was determined by using the calibration gas CCl_4_. A distinct resonance peak of Cl^–^ at 0 eV forms upon electron attachment to CCl_4_ ^38^ under the same experimental conditions. Right after the interaction region a Faraday cup was placed to collect the amount of electron current (about 50 nA). The anions, created in the collision chamber, were extracted by a weak electric field in the direction of the quadrupole mass spectrometer. After the mass analysis, ions were deflected off-axis by 90° with an electrostatic deflector and finally detected by a channeltron. To record the anion efficiency curves shown below, the corresponding mass of the anion was set using the quadrupole and the electron energy was scanned repeatedly in a chosen electron energy range. The onset of the measured ion yield was derived by the method introduced in ref 39.

#### 2.2.2 CLUB Experiment

Electron collisions with microhydrated SCNdMeU were studied using the CLUB setup. CLUB is a complex molecular beam experiment that allows sequential studies of electron- and photon-induced processes on a single, well-defined molecular beam target.^40^ In the present experiment, we used only the TOF part of the setup with a variable-electron-energy gun. Microhydrated molecules were prepared by clustering the precursor SCNdMeU with water in the adiabatic expansion of He buffer gas into vacuum. We describe the methodology in our previous study.^41^

SCNdMeU molecules were sublimed from the solid sample in an oven heated to 120 °C, and water was added to the He buffer gas via a Nafion membrane submerged in a temperature-stabilized water reservoir of a Pergo humidifier. The degree of microhydration was controlled by the He expansion gas pressure, which was set to 1 bar for low hydration conditions, 1.5 bar for medium hydration conditions and 2 bar for high hydration conditions. The beam of microhydrated molecules was crossed by a beam of low-energy electrons at a constant energy. The energy axis was calibrated against the 4.4 eV DEA resonance of O^–^ from CO_2_. However, the instrument’s simple electron gun produces electrons with a broad energy distribution (∼1 eV) and a strongly varying electron current at energies below 1.5 eV, which should be taken into account during data interpretation. These effects were analyzed and described in our publication.^42^ Anions produced by electron collisions with the molecular beam target were analyzed using a custom reflectron TOF spectrometer built by Stefan Kaesdorf GmbH.

Cluster experiments require high sample concentrations in the molecular beam expansion. Therefore, we had to work at temperatures close to thermal decomposition and only over limited time windows. Consequently, solvent effects on the dissociation dynamics were explored only at a single electron energy of 1.5 eV, where the electron gun characteristics are well reproducible. We measured the full 3D spectrum (electron energy, *m*/*z*, and anion intensity) for only one hydration condition.

#### 2.2.3 Computational

The geometries of all species involved in the investigated fragmentation pathways in the CEMB experiment were fully optimized at the M06–2X^43^ level of theory using the aug-cc-pVTZ basis set.^44,45^

The optimal structures of the hydrates were obtained within MD simulations followed by clustering and QM calculations. Previously, such methodology provided us with reasonable geometries and energetics of the studied microsolvates.^46^ First, the conformational scan concerning the rotation of the SCN in the neutral SCNdMeU molecule was performed at the B3LYP^47–49^ /6–31++G­(d,p)^50,51^ level of theory using the PCM solvent model (water),^52^ and the Gaussian16 ^53^ package. The lowest-energy conformer was optimized as an anion radical and parametrized for the Amber force field using AmberTools24 ^54^ with charges and geometry derived from QM calculations. The system was transferred to Gromacs18,^55,56^ solvated with 1 to 6 water molecules, and placed at the center of a cubic box (40 Å). Three replicas with different initial water orientations were generated. Systems were minimized and simulated for 1 μs in the NVT ensemble at 300 K with a 2 fs time step using a modified Berendsen thermostat.^57^ Nonbonded interactions were treated with the Verlet cutoff scheme and a twin-range cutoff of 1.8 nm under periodic boundary conditions.^58^ Bonds involving hydrogen were constrained using LINear Constraint Solver (LINCS).^59,60^ Trajectories were merged, centered, and corrected for translational and rotational motion of the center of mass.

Frames containing a defined number (1–6) of water molecules within 10 Å of SCNdMeU were selected (Table S1) and clustered using the GROMOS method in Gromacs with varying cutoffs to obtain representative structures. Clusters with >10 frames were retained for QM calculations (Table S2).

Electronic energies were used to compute VDE and VAE, and Gibbs free energies for AEA (Tables S3–S8). AEA, VDE, and VAE were calculated as follows:

$$AEA=G_{anion}-G_{neutral}$$

$$VDE=E_{neutral\left(in\;anion\;geometry\right)}-E_{anion}$$

$$VAE=E_{neutral}-E_{anion\left(in\;neutral\;geometry\right)}$$

These QM calculations were performed at the B3LYP/DGDZVP++^61^ level in the gas phase. The DGDZVP++ basis set augments DGDZVP with diffuse sp functions on all atoms, with exponents set to one-third of the smallest original exponent.^62^ The DEA profiles were calculated using fully optimized structures of anionic clusters (see Supporting Information, Figures S4–S9).

The weighted average was calculated using all conformational clusters for which ten or more geometries were identified during the geometry clustering. The population of each cluster obtained from the MD simulations was used as the weighting factor to account for the relative abundance of the conformational states in the equilibrium ensemble.

Regardless of the QM level, M06–2X/aug-cc-pVTZ or B3LYP/DGZVP++, harmonic vibrational frequencies were obtained by diagonalization of the Hessian matrix (second derivatives of the energy with respect to nuclear coordinates). All optimized structures were characterized as true minima (all positive frequencies) or first-order saddle points (single imaginary frequency). Transition states were refined by gradient optimization and subsequently verified by frequency analysis. Intrinsic reaction coordinate (IRC) calculations were performed to confirm that each transition state connects the correct minima.^63,64^ All calculations were carried out using the Gaussian 16 ^46^ program package.

All data were visualized using the GaussView 6 software.^65^

## 3 Results and Discussion

### 3.1 Electron Attachment to Isolated SCNdMeU

Under CEMB conditions, ion yields at *m*/*z* 26, 32, 58, 151, 170, 171, 196, and 197 were detected. The corresponding anion efficiency curves are shown in Figures 1 and S10, whereas the peak maxima and threshold energies are summarized in Tables 1 and S9. For mechanistic clarity, the observed fragmentation products may be divided into two groups: ions formed by direct single-bond cleavage and products whose formation most likely requires additional rearrangement within the transient negative ion. This classification is useful because it shows that, already at the level of the isolated molecule, electron attachment to SCNdMeU leads to a competition between prompt bond-selective dissociation and survival of the parent radical anion.

**Figure 1:**
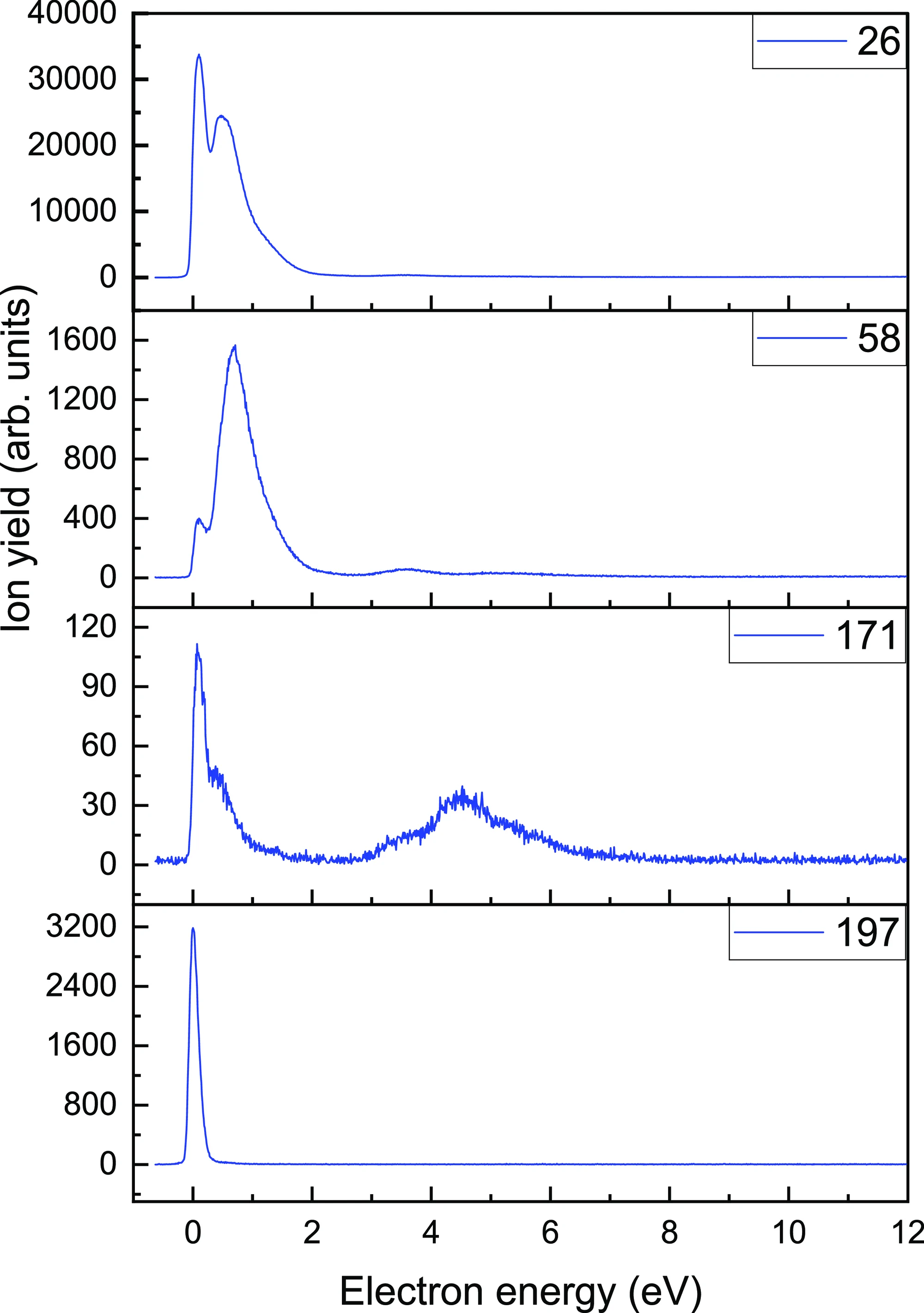
Anion efficiency curves of the fragment anions formed upon electron attachment to SCNdMeU: (*m*/*z* 26) CN^–^, (*m*/*z* 58) SCN^–^, (*m*/*z* 171) [SCNdMeU–CN]^−^, and (*m*/*z* 197) SCNdMeU^●–^.

**Table 1:** A Summary of the Observed Anions is Provided, Detailing Their Masses, Structural Assignments, and Corresponding Peak Values on the Anion Efficiency Curves, along with the Experimental and Calculated Threshold Energies (ΔE_0_)

							Threshold (eV)	
Mass (u)	Anion	Maxima of peak positions (eV)				Max intensivity (Hz)	Expt (∼351 K)	Calcd (ΔE_0_)
**26**	[CN]^−^	∼0	0.5	3.5		33159	∼0	–0.10
**58**	[SCN]^−^	∼0	0.7	3.6	5.2	1525	∼0	0.05
**171**	[SCNdMeU–CN]^−^	3.4	4.4	5.1		35	3.0	1.59
**197**	[SCNdMeU]^●–^	∼0				3138	∼0	–0.92

The appearance of the parent radical anion at *m*/*z* 197 demonstrates that under isolated conditions the anion has a sufficiently long lifetime for detection by mass spectrometry. This observation is fully consistent with the calculated adiabatic electron affinity of 0.92 eV at the M06–2X/aug-cc-pVTZ level, which indicates that vertical electron capture can be followed by structural relaxation to a thermodynamically stable anion radical rather than immediate autodetachment. This point is mechanistically important, because DEA can compete efficiently only when the initially formed transient negative ion survives long enough to couple the excess energy into a dissociative nuclear coordinate.^66^

Besides the parent anion, several fragment anions are formed upon DEA to SCNdMeU. They arise from selective bond cleavage within the thiocyanate substituent or at its linkage to the uracil framework, as illustrated in Figure 2. The observed fragmentation pattern is therefore not random. Instead, it shows that low-energy electron attachment activates a limited set of chemically meaningful reaction coordinates, which is precisely the type of behavior expected for an electron-responsive radiosensitizer scaffold.^67,68^

**Figure 2:**
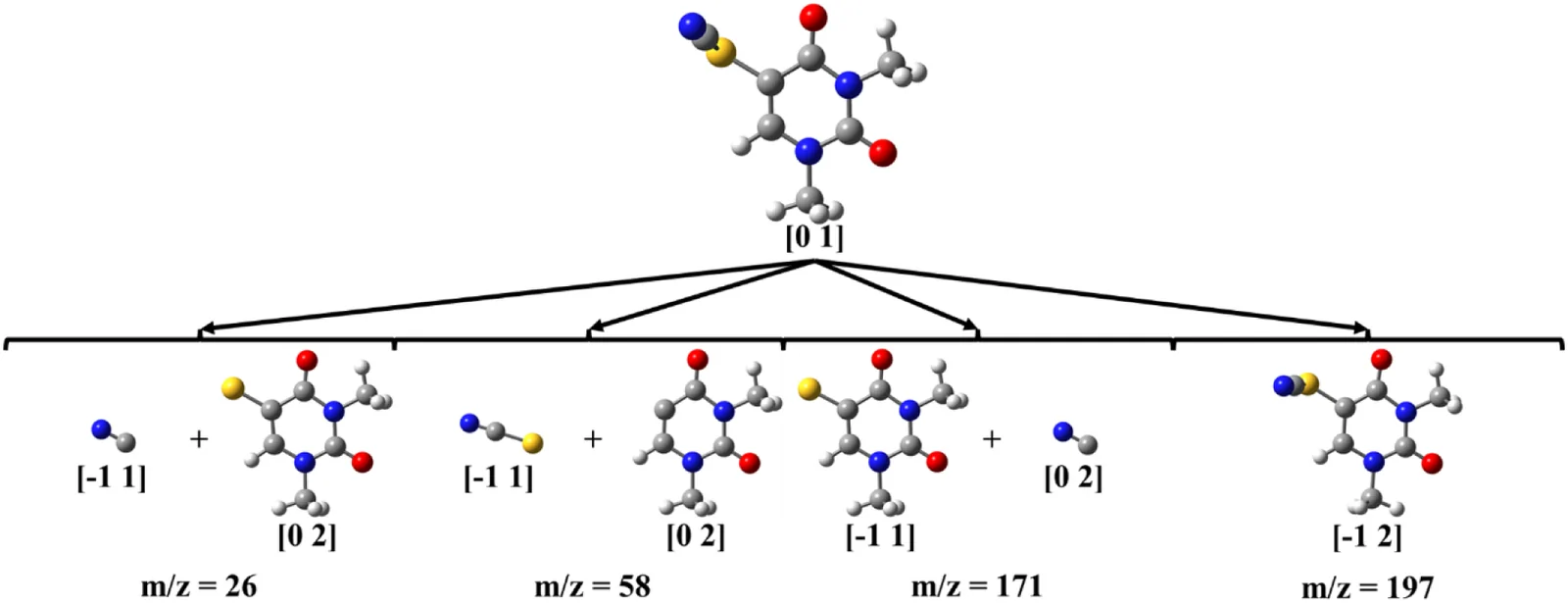
Anion pathways of the SCNdMeU molecule following low-energy electron attachment. The experimentally observed anions are identified by their *m*/*z* values: 26, 58, 171, and 197. For each structure, charge and multiplicity are indicated in brackets. Atoms are color-coded as follows: hydrogen in white, carbon in gray, nitrogen in blue, and sulfur in yellow.

The heaviest fragment anion, detected at *m*/*z* 171, arises from a single-bond cleavage. Specifically, the anion is formed via S–C bond cleavage, with the CN group leaving as a neutral radical fragment, as shown in Figure 2 (*m*/*z* = 171). The dissociative electron attachment reaction can be described as follows:

1 $$e^{-}+\mathrm{SCNdMeU}\to {\mathrm{SCNdMeU}}^{•-}\to {[\mathrm{SCNdMeU-CN}]}^{-}+{\mathrm{CN}}^{•}$$

At the M06–2X/aug-cc-pVTZ level of theory, the calculated reaction energy for neutral SCNdMeU is 1.59 eV. The activation energy for this reaction, corresponding to structure TS_26/171 in Figure 3, lies 0.67 eV above the energy released upon electron attachment (0.92 eV, red dashed line in Figure 3). This explains why the *m*/*z* 171 ion is experimentally observed only as a weak higher-energy channel above 3 eV: the process is electronically accessible, but kinetically much less favorable than the dominant low-energy dissociation pathways. We note that the ion yield at *m*/*z* 171 below the electron energy of 3 eV is an artifact due to the limited mass resolution of the quadrupole (see ion yield for *m*/*z* 170 shown in Figure S10).

**Figure 3:**
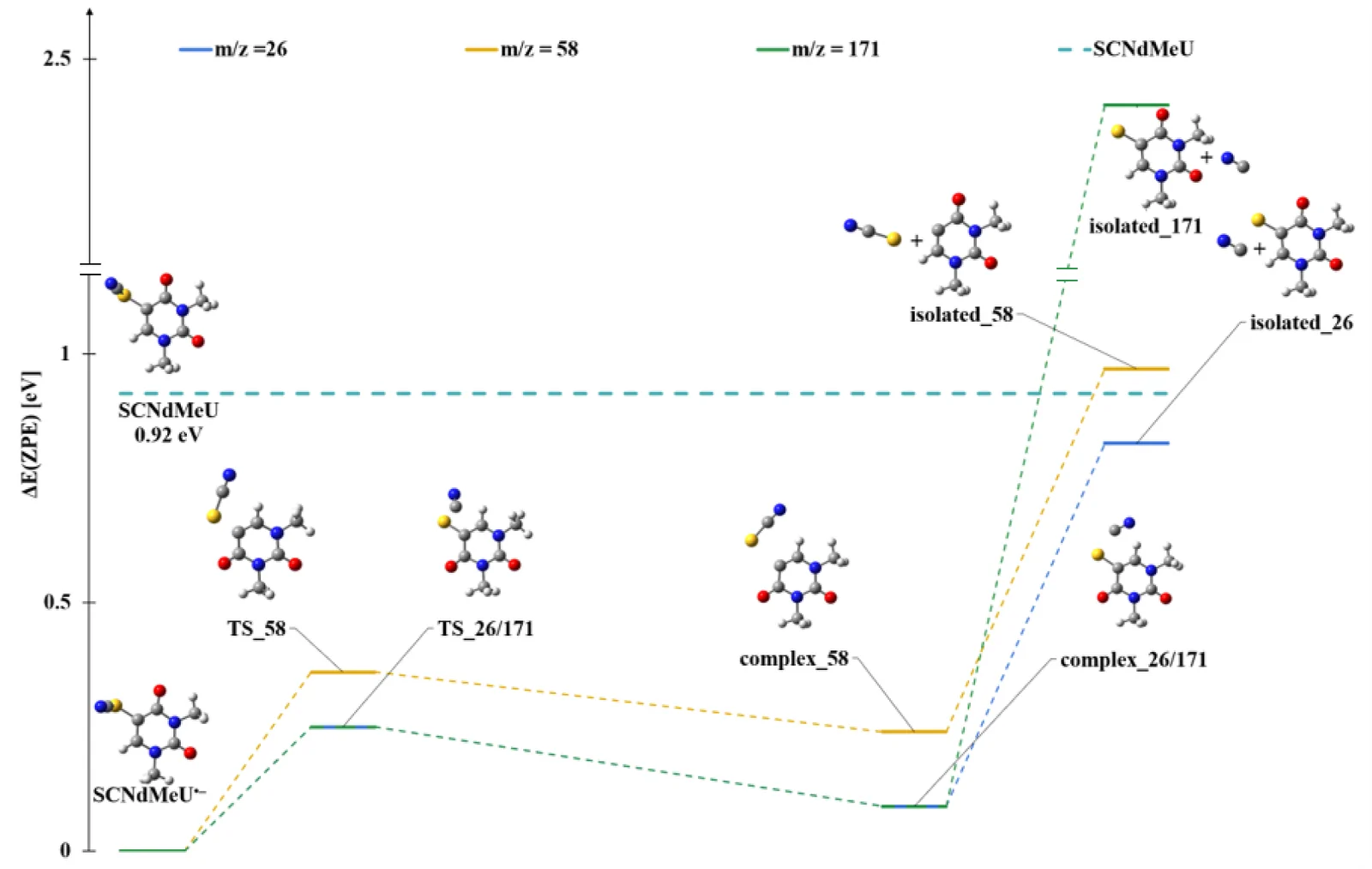
Single bond cleavage pathways, leading from the SCNdMeU^●–^ anion radical to the anionic products at *m*/*z* 26, 58, and 171. TS_x, Complex_x, and Isolated_x, where x refers to the value of *m*/*z*, stand for the transition states, product complex, and isolated monomers respectively.

Another fragment anion produced during DEA to SCNdMeU was detected at *m*/*z* 58. As shown in Figure 2 (*m*/*z* 58), this anion is formed through cleavage of the C5–SCN bond, resulting in the release of SCN^–^. The reaction may be written as

2 $$e^{-}+\mathrm{SCNdMeU}\to {\mathrm{SCNdMeU}}^{•-}\to {[\mathrm{SCNdMeU-SCN}]}^{•}+{\mathrm{SCN}}^{-}$$

The calculated activation energy for cleavage of the C–SCN bond in SCNdMeU^●–^ is 0.36 eV (TS_58 in Figure 3). Thus, the initially formed excited anion retains about 0.56 eV of excess energy above the transition-state level, which makes this reaction accessible at 0.05 eV. At low-electron energies, the SCN^–^ ion yield shows two overlapping features at ∼0 and 0.7 eV. The contribution near zero eV may be ascribed to a hot-band transition.^69^ The abundant detection of SCN^–^ at energies below 2 eV therefore identifies the C5–SCN bond as one of the principal electron-induced dissociation sites in SCNdMeU.

The most intense fragment anion formed during DEA arises from cleavage of the S–CN bond within the thiocyanate substituent. The corresponding reaction can be expressed as

3 $$e^{-}+\mathrm{SCNdMeU}\to {\mathrm{SCNdMeU}}^{•-}\to {[\mathrm{SCNdMeU-CN}]}^{•}+{\mathrm{CN}}^{-}$$

At the M06–2X/aug-cc-pVTZ level, the reaction energy of −0.10 eV predicted for neutral SCNdMeU is consistent with the experimental threshold at ∼0 eV. The activation energy for formation of CN^–^ is only 0.25 eV (TS_26 in Figure 3), which explains why this channel dominates the low-energy part of the spectrum. The strong CN^–^ signal therefore reflects not only favorable thermodynamics, but also a radical-anion potential-energy surface that efficiently directs the excess electron into rupture of the terminal S–CN bond.

Taken together, the relative intensities of the *m*/*z* 26, 58, 171, and 197 ions reveal a clear hierarchy of postattachment processes in isolated SCNdMeU. Electron capture first produces a stable parent radical anion, but this species is not merely a terminal product. Rather, it serves as a branching point from which low-barrier cleavage of the S–CN bond and, to a lesser extent, the C5–SCN bond compete with survival of the intact anion. The much weaker *m*/*z* 171 signal shows that S–C bond cleavage with loss of neutral CN is mechanistically possible but only at higher electron energies above 3 eV. Accordingly, the product distribution is governed primarily by kinetics within the radical-anion manifold rather than by fragment stability alone.

The energy profiles collected in Figure 3 rationalize these experimental trends at the molecular level. Both low-energy channels, formation of CN^–^ and SCN^–^, proceed through relatively small barriers lying below or close to the energy window opened by electron attachment, whereas the pathway leading to *m*/*z* 171 requires substantially more activation. This explains why the parent anion and the two light fragment ions dominate the low-energy part of the spectrum, while the heavier fragment appears only in the higher-energy regime. In other words, the observed ordering of ion yields is encoded directly in the topology of the radical-anion potential-energy surface.

A brief comment is also warranted for the less extensively discussed higher-mass ions, whose detailed characterization is provided in the Supporting Information (Figures S10 and S11, Table S9, Comment S1 and eqs S1–S4). Among the channels discussed in the Supporting Information, the ion at *m*/*z* 170 is particularly noteworthy because it corresponds to HCN loss and is mainly observed below 1 eV, with calculated thresholds ranging from −0.13 to 0.24 eV depending on the structure considered. This result indicates that, besides simple single-bond rupture, the radical-anion manifold of SCNdMeU also allows low-energy pathways involving concerted elimination of a neutral HCN molecule. In contrast, [SCNdMeU–H]^−^ seems not observable within the detection limit of the present apparatus. The calculated thresholds for the candidate structures are clearly positive (1.43–2.64 eV).

Taken together, the behavior of the *m*/*z* 170, 171 and 197 ions further refines the mechanistic picture of electron attachment to isolated SCNdMeU. The dominant outcome remains formation of a bound parent radical anion, whereas fragmentation into higher-mass products samples progressively less favorable regions of the radical-anion potential-energy surface, ranging from kinetically hindered single-bond cleavage (*m*/*z* 171) to low-energy neutral-molecule elimination (*m*/*z* 170).

More broadly, these results identify isolated SCNdMeU as a promising electron-responsive scaffold. The molecule combines a positive adiabatic electron affinity with access to low-barrier dissociation channels that release CN^–^ or SCN^–^ related products immediately after attachment. This combination is highly relevant for radiosensitization, because an effective sensitizer must not only capture low-energy electrons generated during radiolysis but also convert that event into chemically productive bond cleavage.^61,62^ In SCNdMeU, the key electron-induced reactions are already encoded in the isolated-molecule limit, which makes the gas-phase experiment a natural mechanistic reference point for understanding how microsolvation will later modulate, rather than create, the dominant DEA channels.

### 3.2 Electron Attachment to Clusters: SCNdMeU­[H_2_O]_n_

The combined CEMB, CLUB, MD, and quantum-chemical data show that microhydration modulates, but does not extinguish, the reductive activation of SCNdMeU following low-energy electron attachment. In the isolated molecule, DEA opens two chemically relevant channels, cleavage of the S–CN bond and cleavage of the C5–SCN bond, which generate fragment anions and neutral radical counterparts potentially important for subsequent radiation chemistry. This behavior places SCNdMeU within the broader class of electron-responsive radiosensitizer candidates in which selective bond cleavage competes with stabilization of the initially formed radical anion.

Figure 4 captures the onset of this competition under controlled microsolvation conditions. Panel a) of Figure 4 shows the cumulative mass spectrum over the energy range from 0 to 11.5 eV at medium hydration conditions. The cumulative spectrum includes ions detected at all electron energies and therefore can be directly compared to the isolated molecule spectra. The spectrum indicates that hydration stabilizes the radical-anion manifold and thereby shifts the balance between dissociative and nondissociative electron attachment. It is also important to mention that the CLUB setup uses a time-of-flight mass spectrometer with detection times for the parent anion in 10 μs range, while the detection times in the CEMB experiment are longer. These detection times also influence the relative ratio of the parent anion to the fragment anion, following the described anion decay mechanism.^70^ The stabilization of the parent anion upon hydration is supported by the calculated electronic descriptors: the weighted AEA (Tables S3–S8) becomes more negative from −1.31 eV for n = 1 to −1.74 eV for n = 4, while the VDE remains strongly positive throughout the n = 1–6 series (1.82–2.68 eV), consistent with efficient trapping of the excess electron in the hydrated clusters. Thus, the reduced CN–/SCN– signal in Figure 4 reflects solvent-controlled redistribution of postattachment dynamics rather than suppression of attachment itself.

**Figure 4:**
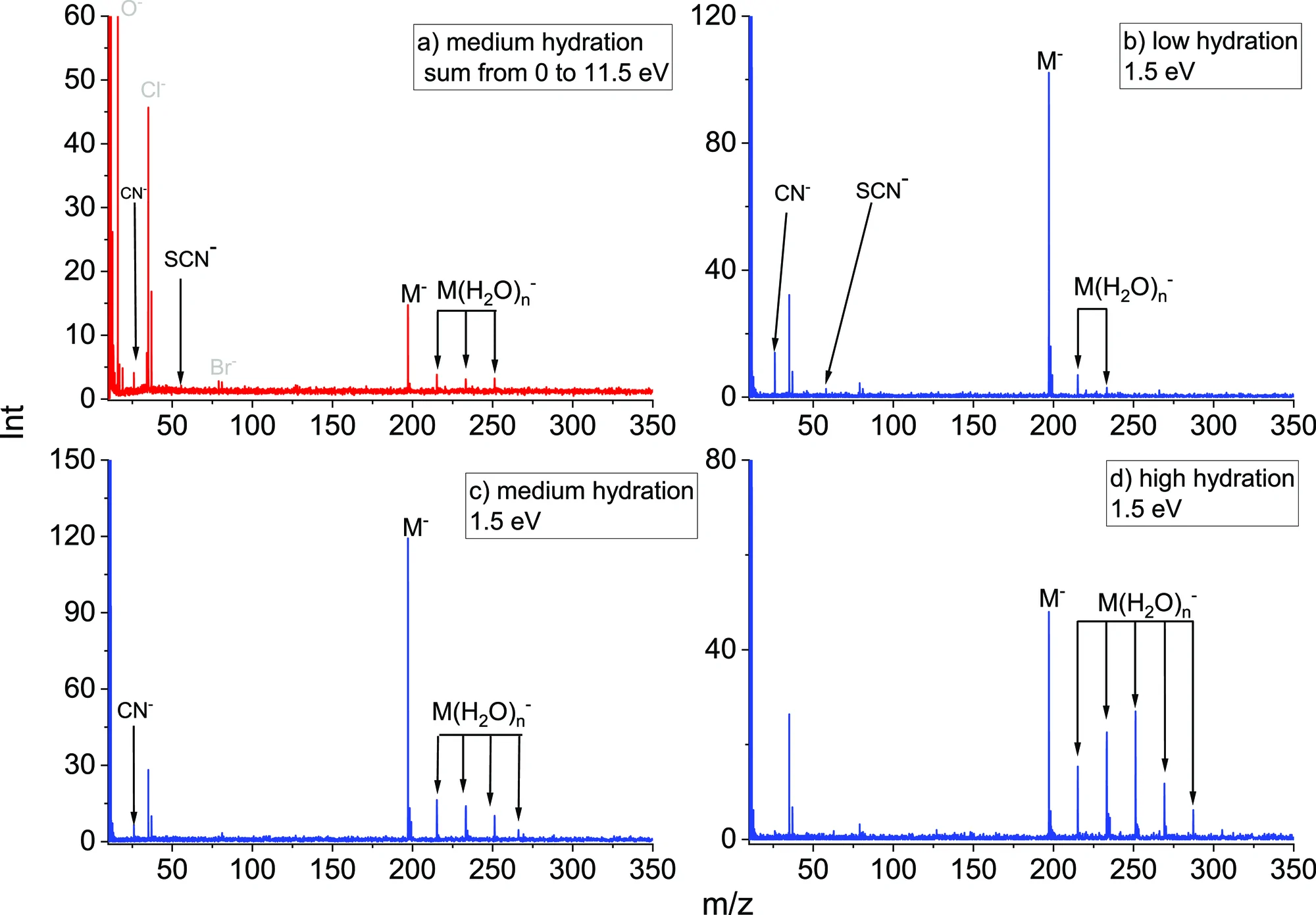
Negative ion mass spectra at 1.5 eV of SCNdMeU upon microhydration obtained as a sum of 47 individual mass spectra measured in the incident electron energy range of 0 eV to 11.5 eV with a constant energy step of 0.25. Panels b) to c) show negative ion mass spectra at constant energy of incident electrons of 1.5 eV at different hydration conditions, from b) low, through c) medium to d) high hydration conditions. Gray labels show assignments for common background peaks; the no-sample background is also shown in SI Figure S12.

Panels b), c) and d) of Figure 4 show the evolution of the individual fragmentation channels with hydration. This comparison is done at a single electron energy to secure stable experimental conditions. Under the driest conditions (Figure 4b), the spectrum contains the parent anion SCNdMeU^–^, the fragment ions CN^–^ and SCN^–^, and weakly hydrated parent-cluster anions SCNdMeU­[H_2_O]_n_^–^ (n = 1–2), showing that electron attachment is followed by direct competition between stabilization of the parent radical anion and dissociation along the S–CN and C5–SCN coordinates. Upon increasing water loading (Figure 4c,d), the fragment-ion intensities decrease while hydrated parent-related ions become increasingly favored, indicating that the first water molecules already redirect the fate of the attached electron away from prompt bond cleavage.

The kinetic barriers (on the free-energy scale) in Figure 5 provide the quantitative basis for this interpretation. For the S–CN cleavage channel, the weighted activation free energy increases overall from 2.31 kcal/mol for n = 1 to 4.22 kcal/mol for n = 6, whereas for C5–SCN cleavage the corresponding values remain consistently higher, varying from 5.89 to 6.50 kcal/mol across the same hydration range (for more detailed results see SI, Tables S10–S15). Hydration therefore preserves the ordering of the two pathways, with S–CN rupture remaining kinetically preferred throughout the microsolvation series, while gradually decreasing the accessibility of both dissociation channels.

**Figure 5:**
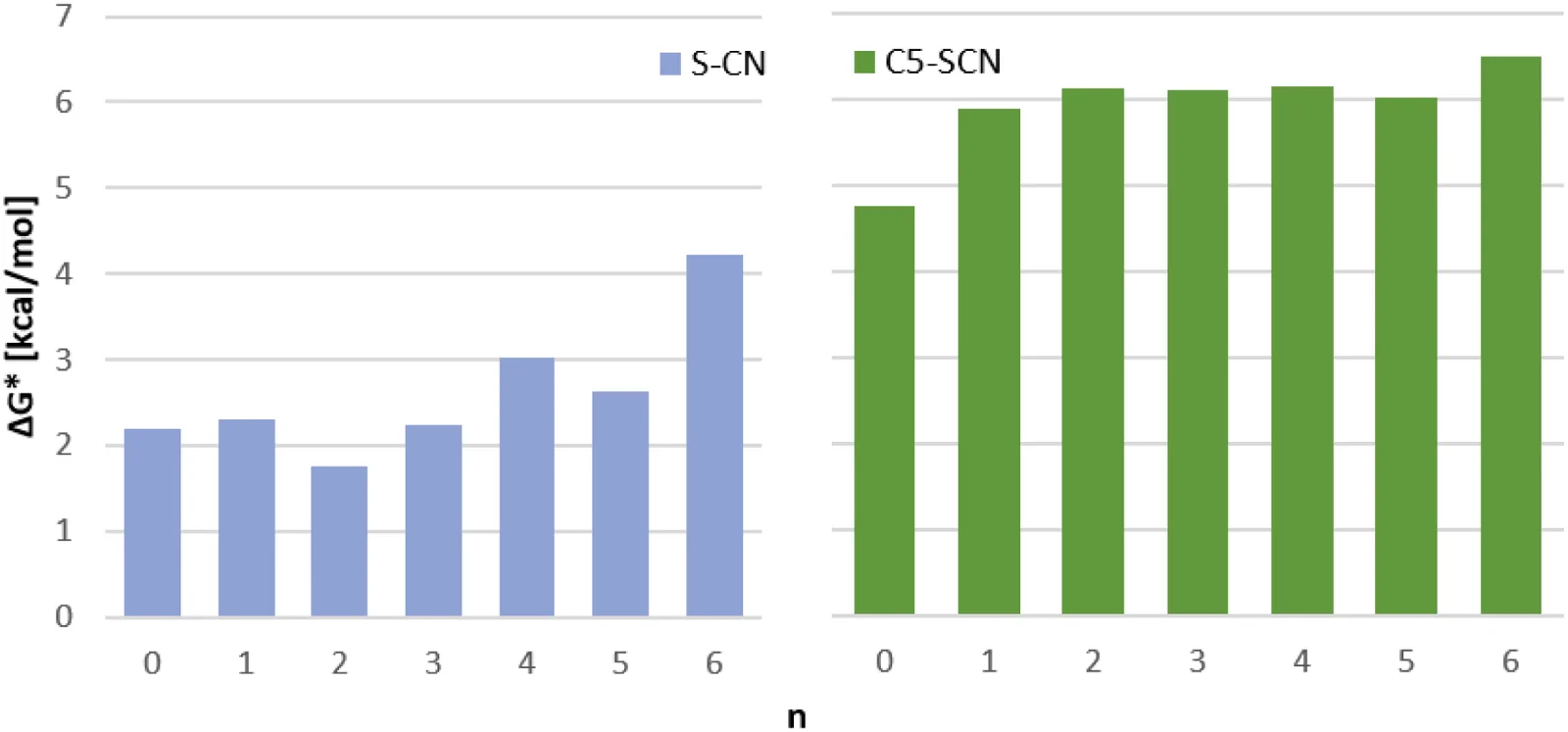
Kinetic barriers for the DEA-induced degradation pathways involving S–CN bond cleavage (left panel) and C5–SCN bond cleavage (right panel), presented on the Gibbs free energy profile, for SCNdMeU­[H_2_O]_n_ clusters with different degrees of hydration (*n* = 1–6, where n denotes the number of water molecules), calculated at the B3LYP/DGDZVP++ level of theory.

The nonmonotonic increase of the calculated barriers suggests that the first water molecules do not act as a purely inhibitory medium. Rather, individual hydration motifs can still support geometries favorable for dissociation, especially at low n, whereas the ensemble-averaged effect of a more developed hydration shell is stabilization of the bound radical anion at the expense of fragment formation. In this sense, Figures 4 and 5 together point to solvent gating of DEA: hydration does not close the reactive channels abruptly, but progressively raises the kinetic cost of accessing them.^40,71^

The MD-based configurational analysis supports this interpretation. For each hydration number, several populated motifs contribute to the observed reactivity, with 6–10 structurally significant cluster families retained for the QM analysis depending on n. The experimental spectra should therefore be viewed as ensemble-averaged observables over multiple local hydrogen-bonding arrangements rather than signatures of a single hydrated structure. This ensemble picture explains why the attenuation of fragmentation in Figure 4 is gradual and channel-dependent rather than abrupt.

Comparison with other modified uridines further clarifies the significance of the present results. In 5-iodo-4-thio-2′-deoxyuridine, low-energy electron attachment efficiently induces deiodination, whereas in 5-bromo-4-thiouracil and 5-bromo-4-thio-2′-deoxyuridine the DEA pattern remains highly sensitive to the detailed molecular scaffold.^30,31^ Classical brominated pyrimidines such as 5-bromouridine likewise owe their radiosensitizing activity to electron-induced bond cleavage and generation of reactive radical intermediates.^72–74^

Against this background, SCNdMeU represents a distinct variant of the same design principle: instead of dehalogenation, its electron-driven chemistry proceeds through S–CN and C5–SCN rupture, while retaining measurable reactivity under microsolvation.

This point is important for the radiosensitization in aqueous media. A useful sensitizer must capture radiation-generated electrons efficiently, retain the resulting radical anion long enough for chemistry to occur, and still preserve access to bond-cleavage channels that generate reactive species.^61,62,75^

SCNdMeU appears to satisfy the first two conditions even in the hydrated regime examined here, whereas the third is moderated, but not abolished, by solvent stabilization. The combined experimental and computational results therefore suggest that water acts as a kinetic buffer rather than a complete quencher of reductive activation.^76–78^

Accordingly, extrapolation to the fully hydrated cellular environment should not lead to the conclusion that SCNdMeU becomes electronically inert. Rather, the present data support a model in which hydration shifts the branching ratio toward parent-radical-anion stabilization while still permitting electron-driven release of CN^–^/SCN^–^ derived reactive species with non-negligible efficiency. This conclusion is consistent with broader concepts of radiosensitizer action involving hydrated or quasi-free electrons in water-rich biological media.^33,61,69,79^

More generally, the present results emphasize that the key descriptor of radiosensitizer performance is not electron affinity alone, but the entire free-energy landscape linking attachment, radical-anion stabilization, and selective bond rupture.^16^

## 4 Conclusions

Low-energy electron attachment to isolated SCNdMeU efficiently forms a bound parent radical anion and opens several dissociative channels, demonstrating that this modified nucleoside is intrinsically susceptible to reductive activation. In the gas phase, the dominant DEA pathways involve cleavage of the S–CN and C5–SCN bonds, whereas the channel leading to *m*/*z* 171 is much less competitive, showing that the postattachment chemistry is governed primarily by the topology of the radical-anion potential-energy surface and the relative kinetic accessibility of individual bond-cleavage coordinates.

The microsolvation study shows that hydration does not suppress electron capture itself, but instead changes the fate of the attached electron. With increasing hydration, the fragment-ion signal decreases while hydrated parent-related anions become increasingly favored, which is consistent with progressive stabilization of the radical-anion manifold. This interpretation is supported by the calculated electronic properties of SCNdMeU­[H_2_O]_n_, for which the weighted AEA becomes more negative from −1.31 eV for *n* = 1 to −1.74 eV for *n* = 4, while the VDE remains strongly positive throughout the hydration series, indicating efficient electron trapping in the clusters.

At the same time, hydration modifies the kinetics of dissociation rather than abolishing reactivity altogether. For the favored S–CN cleavage channel, the weighted activation free energy increases overall from 2.31 kcal/mol for *n* = 1 to 4.22 kcal/mol for *n* = 6, whereas the competing C5–SCN cleavage remains less favorable across the entire series, with weighted barriers of 5.89–6.50 kcal/mol. Thus, water acts as a kinetic gate for DEA: it stabilizes the parent radical anion (even in the largest cluster, SCNdMeU­[H_2_O]_6_, the excess electron remains almost completely localized on the SCNdMeU molecule rather than being distributed across water network, justifying the description of the cluster as a “parent radical anion” hydrated by water molecules) more strongly than the dissociative transition states, thereby shifting the branching ratio toward anion survival while preserving access to the principal fragmentation channels.

These results have direct implications for the radiosensitizing potential of SCNdMeU. An effective electron-driven radiosensitizer must combine efficient electron capture with chemically productive postattachment reactivity, and SCNdMeU satisfies both criteria: it readily binds the excess electron and retains access to bond-cleavage channels capable of generating reactive fragments even under hydrated conditions. More generally, the present work shows that the biologically relevant question is not whether hydration switches DEA on or off, but how solvation redistributes the competition between radical-anion stabilization and selective bond rupture.

## Supplementary Material



## Data Availability

XYZ geometries of all stationary points are deposited in the zenodo.org repository, DOI: 10.5281/zenodo.21703661. The CEMB data can be found at DOI: 10.5281/zenodo.21777878. The CLUB data can be found at DOI:10.5281/zenodo.21978305.

## Notes

Purity characterization of the SCNdMeU sample (Figures S1–S3); microhydration clustering data (Tables S1–S2); calculated electron affinity values (Tables S3–S8) and 3D cluster visualizations (Figures S4–S9); experimental anion efficiency curves and mass spectra for fragment ions (Figures S10–S12, Table S9, and Comment S1); and calculated thermodynamic and kinetic activation barriers for the S–CN^–^ and C–SCN^–^ dissociation channels (Tables S10–S15) (PDF)

## References

[1] Holland J. (2001). New Treatment
Modalities in Radiation Therapy. J. Intraven.
Nurs..

[2] Dasu A., Denekamp J. (1998). New Insights into Factors
Influencing the Clinically
Relevant Oxygen Enhancement Ratio. Radiother.
Oncol..

[3] Rak J., Chomicz L., Wiczk J., Westphal K., Zdrowowicz M., Wityk P., Żyndul M., Makurat S., Golon Ł. (2015). Mechanisms
of Damage to DNA Labeled with Electrophilic Nucleobases Induced by
Ionizing or UV Radiation. J. Phys. Chem. B.

[4] Joiner, M. C. ; Van der Kogel, A. Basic Clinical Radiobiology, 5th ed.; CRC Press: Boca Raton, FL, USA, 2019.

[5] Choy, H. Chemoradiation in Cancer Therapy; Humana Press Inc.: Totowa, NJ, 2011.

[6] Hall, E. J. ; Giaccia, A. J. Radiobiology for the Radiologist; Lippincott Williams & Wilkins: Philadelphia, PA, 2006.

[7] Rockwell S., Dobrucki I. T., Kim E. Y., Marrison S. T., Vu V. T. (2009). Hypoxia
and Radiation Therapy: Past History, Ongoing Research, and Future
Promise. Curr. Mol. Med..

[8] Steenken S. P. B. (1989). Nucleosides,
and Nucleotides: Aqueous Solution Redox Chemistry and Transformation
Reactions of Their Radical Cations and e^–^ and OH
Adducts. Chem. Rev..

[9] Wardman P. (2007). Chemical Radiosensitizers
for Use in Radiotherapy. Clin. Oncol..

[10] Chomicz L., Zdrowowicz M., Kasprzykowski F., Rak J., Buonaugurio A., Wang Y., Bowen K. H. (2013). How to Find Out Whether a 5-Substituted
Uracil Could be a Potential DNA Radiosensitizer. J. Phys. Chem. Lett..

[11] Wang H., Mu X., He H., Zhang X. D. (2018). Cancer Radiosensitizers. Trends Pharmacol. Sci..

[12] Erikson R., Szybalski W. (1963). Molecular Radiobiology of Human Cell
Lines: V. Comparative
Radiosensitizing Properties of 5-Halodeoxycytidines and 5-Halodeoxyuridines. Radiat. Res..

[13] Nabben F. J., Karman J. P., Loman H. (1982). Inactivation of Biologically Active
DNA by Hydrated Electrons. Int. J. Radiat. Biol.
Relat. Stud. Phys., Chem. Med..

[14] Kinsella T. J., Dobson P. P., Mitchell J. B., Fornace A. J. (1987). Enhancement
of X
Ray Induced DNA Damage by Pre-Treatment with Halogenated Pyrimidine
Analogs. Int. J. Radiat. Oncol. Biol. Phys..

[15] Chomicz L., Rak J., Storoniak P. (2012). Electron-Induced
Elimination of the Bromide Anion from
Brominated Nucleobases. A Computational Study. J. Phys. Chem. B.

[16] Spisz P., Zdrowowicz M., Makurat S., Kozak W., Skotnicki K., Bobrowski K., Rak J. (2019). Why Does the Type of Halogen Atom
Matter for the Radiosensitizing Properties of 5-Halogen Substituted
4-Thio-2′-Deoxyuridines?. Molecules.

[17] Li X., Sanche L., Sevilla M. D. (2002). Dehalogenation
of 5-Halouracils after
Low Energy Electron Attachment: A Density Functional Theory Investigation. J. Phys. Chem. A.

[18] Wetmore S. D., Boyd R. J., Eriksson L. A. (2001). A Theoretical Study of 5-Halouracils:
Electron Affinities, Ionization Potentials and Dissociation of the
Related Anions. Chem. Phys. Lett..

[19] Watanabe R., Nikjoo H. (2002). Modeling the Effect
of Incorporated Halogenated Pyrimidine
on Radiation-Induced DNA Strand Breaks. Int.
J. Radiat. Biol..

[20] Chapman J. D., Greenstock C. L., Reuvers A. P., McDonald E., Dunlop I. (1973). Radiation
Chemical Studies with Nitrofurazone as Related to Its Mechanism of
Radiosensitization. Radiat. Res..

[21] Michael B. D., O’neill P. (2000). Molecular
biology. A sting in the tail of electron
tracks. Science.

[22] von Sonntag, C. Free-Radical-Induced DNA Damage and Its Repair: A Chemical Perspective; Springer Science & Business Media: Berlin, Germany, 2006.

[23] Dunn D. B., Smith J. D. (1954). Incorporation of Halogenated Pyrimidines into the Deoxyribonucleic
Acids of Bacterium Coli and Its Bacteriophages. Nature.

[24] Brust D., Feden J., Farnsworth J., Amir C., Broaddus W., Valerie K. (2000). Radiosensitization
of Rat Glioma with Bromodeoxycytidine
and Adenovirus Expressing Herpes Simplex Virus-Thymidine Kinase Delivered
by Slow, Rate-Controlled Positive Pressure Infusion. Cancer Gene Ther..

[25] Cecchini S., Girouard S., Huels M. A., Sanche L., Hunting D. J. (2004). Single-Strand-Specific
Radiosensitization of DNA by Bromodeoxyuridine. Radiat. Res..

[26] Golon Ł., Chomicz L., Rak J. (2014). Electron-Induced Single Strand Break
in the Nucleotide of 5- and 6-Bromouridine. A DFT Study. Chem. Phys. Lett..

[27] Dextraze M. E., Wagner J. R., Hunting D. J. (2007). 5-Bromodeoxyuridine
Radiosensitization:
Conformation-Dependent DNA Damage. Biochemistry.

[28] Lawrence T. S., Davis M. A., Maybaum J., Stetson P. L., Ensminger W. D. (1990). The Effect
of Single versus Double-Strand Substitution on Halogenated Pyrimidine-Induced
Radiosensitization and DNA Strand Breakage in Human Tumor Cells. Radiat. Res..

[29] Makurat S., Chomicz-Mańka L., Rak J. (2016). Electrophilic 5-Substituted Uracils
as Potential Radiosensitizers: A Density Functional Theory Study. ChemPhyschem.

[30] Izadi F., Szczyrba A., Datta M., Ciupak O., Demkowicz S., Rak J., Denifl S. (2023). Electron-Induced
Decomposition of 5-Bromo-4-Thiouracil
and 5-Bromo-4-Thio-2′-Deoxyuridine: The Effect of the Deoxyribose
Moiety on Dissociative Electron Attachment. Int. J. Mol. Sci..

[31] Saqib M., Arthur-Baidoo E., Izadi F., Szczyrba A., Datta M., Demkowicz S., Rak J., Denifl S. (2023). Dissociative Electron
Attachment to 5-Iodo-4-Thio-2′-Deoxyuridine: A Potential Radiosensitizer
of Hypoxic Cells. J. Phys. Chem. Lett..

[32] Lochmann C., Luxford T. F. M., Makurat S., Pysanenko A., Kočišek J., Rak J., Denifl S. (2022). Low-Energy
Electron Induced Reactions in Metronidazole at Different Solvation
Conditions. Pharmaceuticals.

[33] Westphal K., Wiczk J., Miloch J., Kciuk G., Bobrowski K., Rak J. (2015). Irreversible Electron Attachment - a Key to DNA Damage by Solvated
Electrons in Aqueous Solution. Org. Biomol.
Chem..

[34] Kumar A., Sevilla M. D. (2019). Reaction of Electrons
with DNA: Radiation Damage to
DNA. Chem. Rev..

[35] Zdrowowicz M., Datta M., Rychłowski M., Rak J. (2022). Radiosensitization
of PC3 Prostate Cancer Cells by 5-Thiocyanato-2′-deoxyuridine. Cancers.

[36] Noikham M., Yotphan S. (2019). Copper-Catalyzed Regioselective Direct C–H Thiolation
and Thiocyanation of Uracils. Eur. J. Org. Chem..

[37] Saqib M., Izadi F., Isierhienrhien L. U., Ončák M., Denifl S. (2023). Decomposition of Triazole and 3-Nitrotriazole
upon
Low-Energy Electron Attachment. Phys. Chem.
Chem. Phys..

[38] Pelc A. (2022). Dissociative
Electron Attachment to Carbon Tetrachloride Molecules. Acta Phys. Pol., A.

[39] Meißner R., Feketeová L., Bayer A., Postler J., Limão-Vieira P., Denifl S. (2019). Positive and Negative Ions of the
Amino Acid Histidine
Formed in Low-Energy Electron Collisions. J.
Mass Spectrom..

[40] Fárník M., Fedor J., Kočišek J., Lengyel J., Pluhařová E., Poterya V., Pysanenko A. (2021). Pickup and
Reactions of Molecules on Clusters Relevant for Atmospheric and Interstellar
Processes. Phys. Chem. Chem. Phys..

[41] Kočišek J., Pysanenko A., Fárník M., Fedor J. (2016). Microhydration
Prevents Fragmentation of Uracil and Thymine by Low-Energy Electrons. J. Phys. Chem. Lett..

[42] Kočišek J., Grygoryeva K., Lengyel J., Fárník M., Fedor J. (2016). Effect of
Cluster Environment on the Electron Attachment to 2-Nitrophenol. Eur. Phys. J. D.

[43] Zhao Y., Truhlar D. G. (2008). The M06 Suite of
Density Functionals for Main Group
Thermochemistry, Thermochemical Kinetics, Noncovalent Interactions,
Excited States, and Transition Elements: Two New Functionals and Systematic
Testing of Four M06-Class Functionals and 12 Other Functionals. Theor. Chem. Acc..

[44] Godbout N., Salahub D. R., Andzelm J., Wimmer E. (1992). Optimization
of Gaussian-Type
Basis Sets for Local Spin Density Functional Calculations. Part I.
Boron through Neon, Optimization Technique and Validation. Can. J. Chem..

[45] Sosa C., Andzelm J., Elkin B. C., Wimmer E., Dobbs K. D., Dixon D. A. (1992). A Local Density
Functional Study of the Structure and
Vibrational Frequencies of Molecular Transition-Metal Compounds. J. Phys. Chem..

[46] Makurat S., Yuan Q., Czub J., Chomicz-Mańka L., Cao W., Wang X.-B., Rak J. (2022). Guanosine
Dianions Hydrated by One
to Four Water Molecules. J. Phys. Chem. Lett..

[47] Becke A. D. (1988). Density-Functional
Exchange-Energy Approximation with Correct Asymptotic Behavior. Phys. Rev. A.

[48] Becke A. D. (1993). Density-functional
thermochemistry. III. The Role of Exact Exchange. J. Chem. Phys..

[49] Lee C., Yang W., Parr R. G. (1988). Development
of the Colle-Salvetti
Correlation-Energy Formula into a Functional of the Electron Density. Phys. Rev. B.

[50] Ditchfield R., Hehre W. J., Pople J. A. (1971). Self-consistent molecular-orbital
methods. IX. An Extended Gaussian-Type Basis for Molecular-Orbital
Studies of Organic Molecules. J. Chem. Phys..

[51] Hehre W. J., Ditchfield R., Pople J. A. (1972). Self—Consistent Molecular
Orbital Methods. XII. Further Extensions of Gaussian—Type Basis
Sets for Use in Molecular Orbital Studies of Organic Molecules. J. Chem. Phys..

[52] Tomasi J., Mennucci B., Cammi R. (2005). Quantum Mechanical
Continuum Solvation
Models. Chem. Rev..

[53] Frisch, M. J. ; Trucks, G. W. ; Schlegel, H. B. ; Scuseria, G. E. ; Robb, M. A. ; Cheeseman, J. R. ; Scalmani, G. ; Barone, V. ; Petersson, G. A. ; Nakatsuji, H. , Gaussian 16, Revision C.01; Gaussian Inc.: Wallingford, CT, 2016.

[54] Case D. A., Aktulga H. M., Belfon K., Cerutti D. S., Cisneros G. A., Cruzeiro V. W. D., Forouzesh N., Giese T. J., Götz A. W., Gohlke H. (2023). AmberTools. J. Chem. Inf. Model..

[55] Abraham M. J., Murtola T., Schulz R., Páll S., Smith J. C., Hess B., Lindahl E. (2015). GROMACS: High Performance
Molecular Simulations through Multi-Level Parallelism from Laptops
to Supercomputers. SoftwareX.

[56] Páll, S. ; Abraham, M. J. ; Kutzner, C. ; Hess, B. ; Lindahl, E. Tackling Exascale Software Challenges in Molecular Dynamics Simulations with GROMACS. In Solving Software Challenges for Exascale, Markidis, S. ; Laure, E. , Eds.; Springer: Cham, Switzerland, 2015; Vol. 8759, pp. 3–27

[57] Berendsen H. J. C., Postma J. P. M., van
Gunsteren W. F., DiNola A., Haak J. R. (1984). Molecular
Dynamics with Coupling to an External Bath. J. Chem. Phys..

[58] Páll S., Hess B. (2013). A Flexible Algorithm for Calculating Pair Interactions on SIMD Architectures. Comput. Phys. Commun..

[59] Hess B., Bekker H., Berendsen H. J. C., Fraaije J. G. E. M. (1997). LINCS: A Linear
Constraint Solver for Molecular Simulations. J. Comput. Chem..

[60] Hess B. (2008). P-LINCS: A
Parallel Linear Constraint Solver for Molecular Simulation. J. Chem. Theory Comput..

[61] Yurieva A. G., Poleshchuk O. K., Filimonov V. D. (2008). Comparative Analysis of a Full-Electron
Basis Set and Pseudopotential for the Iodine Atom in DFT Quantum-Chemical
Calculations of Iodine-Containing Compounds. J. Struct. Chem..

[62] Rak J., Gutowski M., Dokurno P., Thanh H. V., Błaźejowski J. (1994). Theoretical
Studies on Structure, Thermochemistry, Vibrational Spectroscopy, and
Other Features of ZrX^2–^
_6_ (X = F, Cl,
Br, I): Coulombic Energy in Inorganic and Organic Hexahalogenozirconates. J. Chem. Phys..

[63] Fukui K. (1981). The Path of
Chemical Reactions - The IRC Approach. Acc.
Chem. Res..

[64] Hratchian, H. P. ; Schlegel, H. B. F. M. Transition States, and Following Reaction Pathways on ab Initio Potential Energy Surfaces. In Theory and Applications of Computational Chemistry: the First Forty Years; Dykstra, C. E. ; Frenking, G. ; Kim, K. S. ; Scuseria, G. Eds.; Elsevier Science: New York, 2005; pp. 195–249

[65] Dennington, R. ; Keith, T. ; Millam, J. GaussView, Version 6.1.1.; Semichem Inc., Shawnee Mission KS, 2019.

[66] Sedmidubská B., Kočišek J. (2024). Interaction of Low-Energy Electrons
with Radiosensitizers. Phys. Chem. Chem. Phys..

[67] Kumar S., Sarma M. (2024). Dissociative Electron
Attachment to Halogenated Nucleotides: a Quest
for Better Radiosensitizers. Phys. Chem. Chem.
Phys..

[68] Kumar A., Becker D., Adhikary A., Sevilla M. D. (2019). Reaction of Electrons
with DNA: Radiation Damage to Radiosensitization. Int. J. Mol. Sci..

[69] Hahndorf I., Illenberger E., Lehr L., Manz J. (1994). Temperature Effects
of Dissociative Electron Attachment to CF_3_Cl. Chem. Phys. Lett..

[70] Izadi F., Luxford T. F. M., Sedmidubská B., Arthur-Baidoo E., Kočišek J., Ončák M., Denifl S. (2024). Dissociative Electron Attachment Dynamics of a Promising
Cancer Drug Indicates Its Radiosensitizing Potential. Angew. Chem., Int. Ed..

[71] Fabrikant I. I. (2018). Electron
Attachment to Molecules in a Cluster Environment: Suppression and
Enhancement Effects. Eur. Phys. J. D.

[72] Abdoul-Carime H., Limão-Vieira P., Gohlke S., Petrushko I., Mason N. J., Illenberger E. (2004). Sensitization of 5-Bromouridine by
Slow Electrons. Chem. Phys. Lett..

[73] Li Z., Cloutier P., Sanche L., Wagner J. R. (2011). Low-Energy Electron-Induced
Damage in a Trinucleotide Containing 5-Bromouracil. J. Phys. Chem. B.

[74] Greenstock C. L., Whitehouse R. P. (1992). Radiation
Chemical Studies of Sensitization by 5-Bromouridine-5′-Monophosphate
(5-BrUMP). Radiat. Environ. Biophys..

[75] Zdrowowicz, M. ; Chomicz-Mańka, L. ; Butowska, K. ; Spisz, P. ; Falkiewicz, K. ; Czaja, A. ; Rak, J. DNA Damage Radiosensitizers Geared Towards Hydrated Electrons. In Practical Aspects of Computational Chemistry V; Leszczynski, J. ; Shukla, M. K. Eds.; Springer: Cham, Switzerland, 2021; pp. 125–169

[76] McAllister M., Kazemigazestane N., Henry L. T., Gu B., Fabrikant I., Tribello G. A., Kohanoff J. (2019). Solvation Effects on Dissociative
Electron Attachment to Thymine. J. Phys. Chem.
B.

[77] Sedmidubská B., Denisov S., Mostafavi M., Denifl S., Izadi F., Ončák M., Luxford T. F. M., Chvátil D., Pinkas J., Kočišek J. (2025). Nitro-Group
π
System Drives the Interaction of RRx-001 with Electrons in Solution. Chem. -Eur. J..

[78] Poštulka J., Slavíček P., Fedor J., Fárník M., Kočišek J. (2017). Energy Transfer in Microhydrated
Uracil, 5-Fluorouracil, and 5-Bromouracil. J.
Phys. Chem. B.

[79] Ma J., Bahry T., Denisov S. A., Adhikary A., Mostafavi M. (2021). Quasi-Free
Electron-Mediated Radiation Sensitization by C5-Halopyrimidines. J. Phys. Chem. A.

