# Supplementary material for: Dissociative Electron Attachment in 1,3-Dimethyl-5-Thiocyanatouracil at Different Hydration Conditions
Source: J Phys Chem B. 2026 Oct 26;130(44):11768–78. doi: 10.1021/acs.jpcb.6c05644 (PMC13746783; doi:10.1021/acs.jpcb.6c05644)
Supplement: Supplementary file 1 [file jp6c05644_si_001.pdf]

Supporting Information

Dissociative Electron Attachment in 1,3-Dimethyl-5-Thiocyanatouracil at Different Hydration Conditions

Adrian Szczyrba[1], Samanta Makurat[1], Vy T. T. Nguyen[2], Jiakuan Chen[2], Magdalena Datta[1,3], Karol Biernacki[4], Jaroslav Kočišek[5], Jozef Ďurana[5,6], Barbora Sedmidubská[5,7], Sebastian Demkowicz[4], Stephan Denifl[2], Janusz Rak[1]*

[1]Laboratory of Biological Sensitizers, Physical Chemistry Department, Faculty of Chemistry, University of Gdańsk, 80-308 Gdańsk, Poland

[2]Institut für Ionenphysik und Angewandte Physik and Center for Biomolecular Sciences Innsbruck, Universität Innsbruck, Technikerstrasse 25, A-6020 Innsbruck, Austria[2]

[3]Department of Inorganic Chemistry, Faculty of Chemistry, Gdańsk University of Technology, Narutowicza 11/12, 80-233 Gdańsk, Poland

[4]Department of Organic Chemistry, Faculty of Chemistry, Gdańsk University of Technology, Narutowicza 11/12, 80-233 Gdańsk, Poland

[5]Heyrovský Institute of the Czech Academy of Sciences, v.v.i., Dolejškova 3, 18200 Prague, Czech Republic

[6]University of Chemistry and Technology Prague, Technická 5, 166 28 Prague 6, Czech Republic

[7]Institute of Physics of the Czech Academy of Sciences, v.v.i., Na Slovance 1999/2, 182 00 Prague 8, Czech Republic

*Correspondence: janusz.rak@ug.edu.pl

Table of Contents

Figure S1. The chromatogram of SCNdMeU sample (purity = 100%). HPLC analyses were determined by reversed-phase RP-HPLC high-performance liquid chromatography, using a Dionex Ultimate 3000 chromatograph (Thermo Scientific) and a Wakopak Handy ODS analytical column (150 x 4.6 mm). The mobile phase was a system of solvents A and B, applied in a 20-minutes gradient of 0-70% phase B, where: A - 0.1% HCOOH and 0.1% ACN in water; B - 80% ACN. The volumetric flow rate of eluents was 1 mL/min. Detection was carried out at a wavelength of 280 nm. 4

Figure S2. The MS spectrum of SCNdMeU samples registered in positive ionization mode. Theoretical mass: $[M+1]^+$ = 198.0331 Da; Experimental mass: [M+1]+ = 198.0759 Da. The HRMS and MS/MS measurements were performed with using of TripleTOF 5600+ (SCIEX, Framingham, MA, USA) high-resolution mass spectrometer coupled to UPLC system (Nexera X2, Shimadzu, Canby, OR, USA). 5

Figure S3. The 1H NMR spectrum of SCNdMeU sample. NMR spectra were measured in chloroform-d using 500 MHz spectrometer (Varian Unity Inova). 6

Table S1. Number of frames (out of 2703 total) containing 1, 2, 3, 4, 5 and 6 in a 10 Å water shell surrounding SCNdMeU. 7

Table S2. Clustering results of SCNdMeU$[H_2O]_n$, the cutoff used and number of clusters......7

Table S3. The AEA, VEA and VDE of SCNdMeU$[H_2O]_1$. 8

Table S4. The AEA, VEA and VDE of SCNdMeU$[H_2O]_2$. 8

Table S5. The AEA, VEA and VDE of SCNdMeU$[H_2O]_3$. 9

Table S6. The AEA, VEA and VDE of SCNdMeU$[H_2O]_4$. 9

Table S7. The AEA, VEA and VDE of SCNdMeU$[H_2O]_5$. 10

Table S8. The AEA, VEA and VDE of SCNdMeU$[H_2O]_6$. 10

Figure S4. Visualization of SCNdMeU$[H_2O]_1^{\bullet-}$ structures hydrated with one water molecule, optimized at B3LYP/DGDZVP++ level, along with their names and relative cluster population. 11

Figure S5. Visualization of SCNdMeU$[H_2O]_2^{\bullet-}$ structures hydrated with two water molecules, optimized at B3LYP/DGDZVP++ level, along with their names and relative cluster population. 11

Figure S6. Visualization of SCNdMeU$[H_2O]_3^{\bullet-}$ structures hydrated with three water molecules, optimized at B3LYP/DGDZVP++ level, along with their names and relative cluster population. 12

Figure S7. Visualization of SCNdMeU$[H_2O]_4^{\bullet-}$ structures hydrated with four water molecules, optimized at B3LYP/DGDZVP++ level, along with their names and relative cluster population. 13

Figure S8. Visualization of SCNdMeU$[H_2O]_5^{\bullet-}$ structures hydrated with five water molecules, optimized at B3LYP/DGDZVP++ level, along with their names and relative cluster population. 14

Figure S9. Visualization of SCNdMeU$[H_2O]_6^{\bullet-}$ structures hydrated with six water molecules, optimized at B3LYP/DGDZVP++ level, along with their names and relative cluster population. 14

Figure S10. Anion efficiency curves of the fragment anions formed upon electron attachment to SCNdMeU: (m/z 32) $S^{\bullet-}$, (m/z 151) SCN^{-}, (m/z 170) SCNdMeUa-$HCN^{\bullet-}$. The anion efficiency curve for m/z 196 is also included (see comment S1). 15

Table S9. A summary of the observed fragment anions is provided, detailing their masses, structural assignments, and corresponding peak values on the anion efficiency curves, along with the experimental and calculated threshold energies (ΔE_0). 16

COMMENT S1..16

Figure S11. The experimentally observed fragments are identified by their m/z values: 32, 151 and 170. The detected anion signal at m/z 196 may not be ascribed to $[SCNdMeU\text{-}H]^{-}$, see comment S1. For each structure, charge and multiplicity are indicated in brackets. Atoms are color-coded as follows: hydrogen in white, carbon in gray, nitrogen in blue, and sulfur in yellow..18

Figure S12. Negative ion MS at incident electron energy of 1.5 eV measured before hydration dependence spectra panels b),c),d) of the Figure 4 in main article...18

Table S10. Thermodynamic and kinetic barriers of the DEA process (kcal/mol) for the two reaction pathways (S–CN^- and C–SCN^-), calculated at the B3LYP/DGDZVP++ level of theory for $SCNdMeU[H_2O]_1^{\bullet-}$. The highest and lowest ΔG^* values are marked red and green respectively in all the following tables. 19

Table S11. Thermodynamic and kinetic barriers of the DEA process (kcal/mol) for the two reaction pathways (S–CN^- and C–SCN^-), calculated at the B3LYP/DGDZVP++ level of theory for $SCNdMeU[H_2O]_2^{\bullet-}$..19

Table S12. Thermodynamic and kinetic barriers of the DEA process (kcal/mol) for the two reaction pathways (S–CN^- and C–SCN^-), calculated at the B3LYP/DGDZVP++ level of theory for $SCNdMeU[H_2O]_3^{\bullet-}$..20

Table S13. Thermodynamic and kinetic barriers of the DEA process (kcal/mol) for the two reaction pathways (S–CN^- and C–SCN^-), calculated at the B3LYP/DGDZVP++ level of theory for $SCNdMeU[H_2O]_4^{\bullet-}$..20

Table S14. Thermodynamic and kinetic barriers of the DEA process (kcal/mol) for the two reaction pathways (S–CN^- and C–SCN^-), calculated at the B3LYP/DGDZVP++ level of theory for $SCNdMeU[H_2O]_5^{\bullet-}$..21

Table S15. Thermodynamic and kinetic barriers of the DEA process (kcal/mol) for the two reaction pathways (S–CN^- and C–SCN^-), calculated at the B3LYP/DGDZVP++ level of theory for $SCNdMeU[H_2O]_6^{\bullet-}$..21

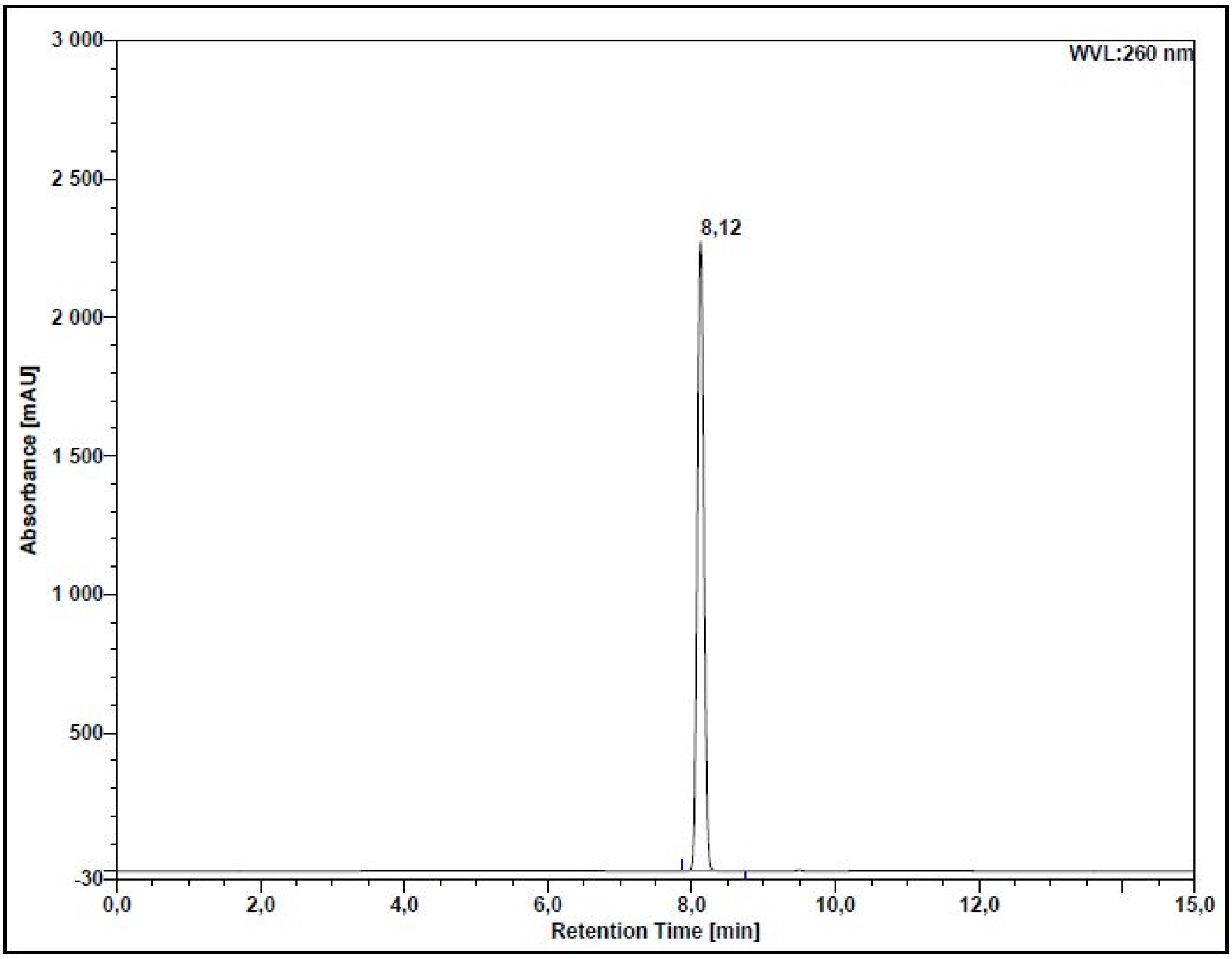

Figure S1. The chromatogram of SCNdMeU sample (purity = 100%). HPLC analyses were determined by reversed-phase RP-HPLC high-performance liquid chromatography, using a Dionex Ultimate 3000 chromatograph (Thermo Scientific) and a Wakopak Handy ODS analytical column (150 x 4.6 mm). The mobile phase was a system of solvents A and B, applied in a 20-minutes gradient of 0-70% phase B, where: A - 0.1% HCOOH and 0.1% ACN in water; B - 80% ACN. The volumetric flow rate of eluents was 1 mL/min. Detection was carried out at a wavelength of 280 nm.

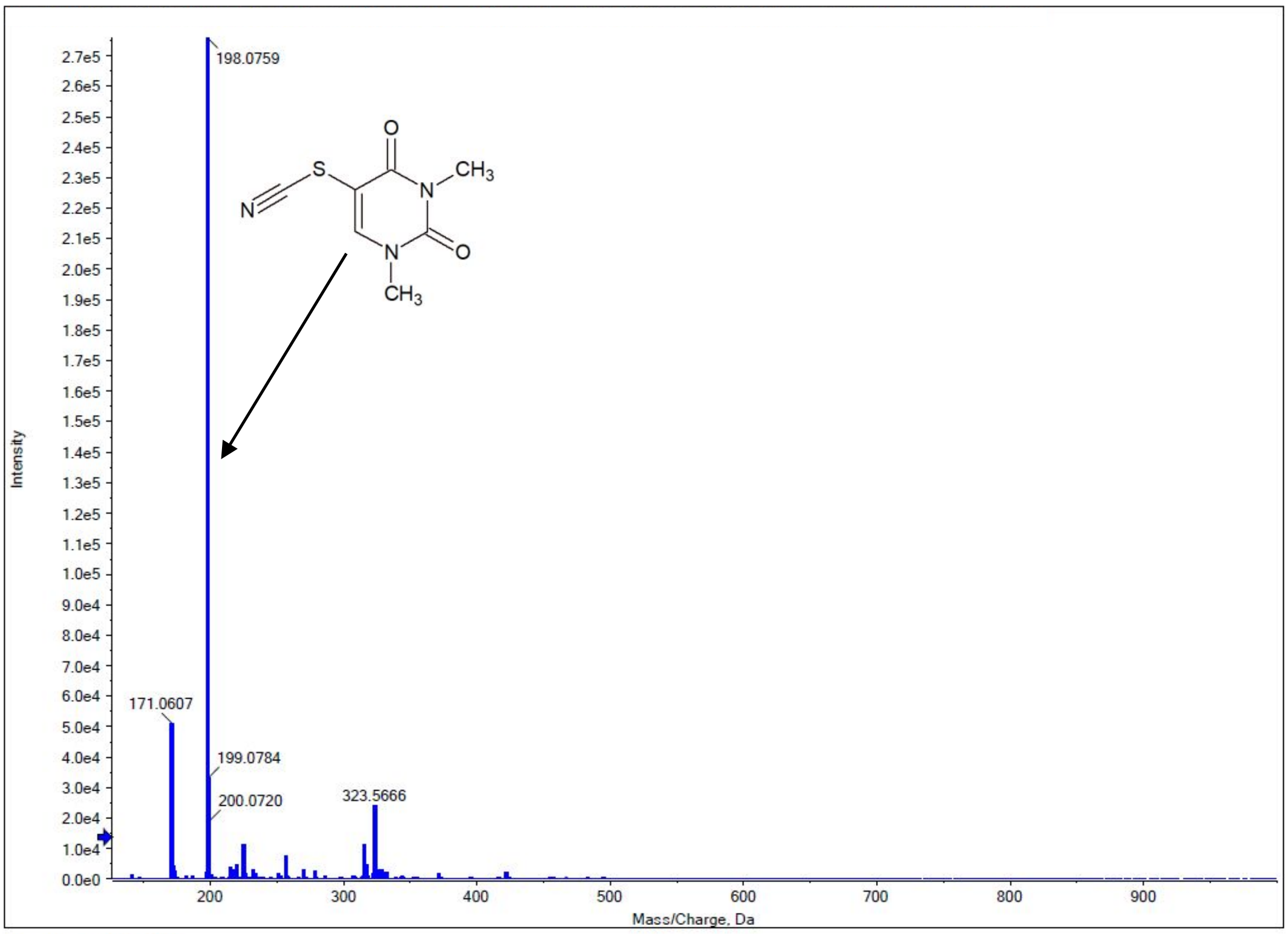

Figure S2. The MS spectrum of SCNdMeU samples registered in positive ionization mode. Theoretical mass: $[M+1]^{+}$ = 198.0331 Da; Experimental mass: [M+1]+ = 198.0759 Da. The HRMS and MS/MS measurements were performed with using of TripleTOF 5600+ (SCIEX, Framingham, MA, USA) high-resolution mass spectrometer coupled to UPLC system (Nexera X2, Shimadzu, Canby, OR, USA).

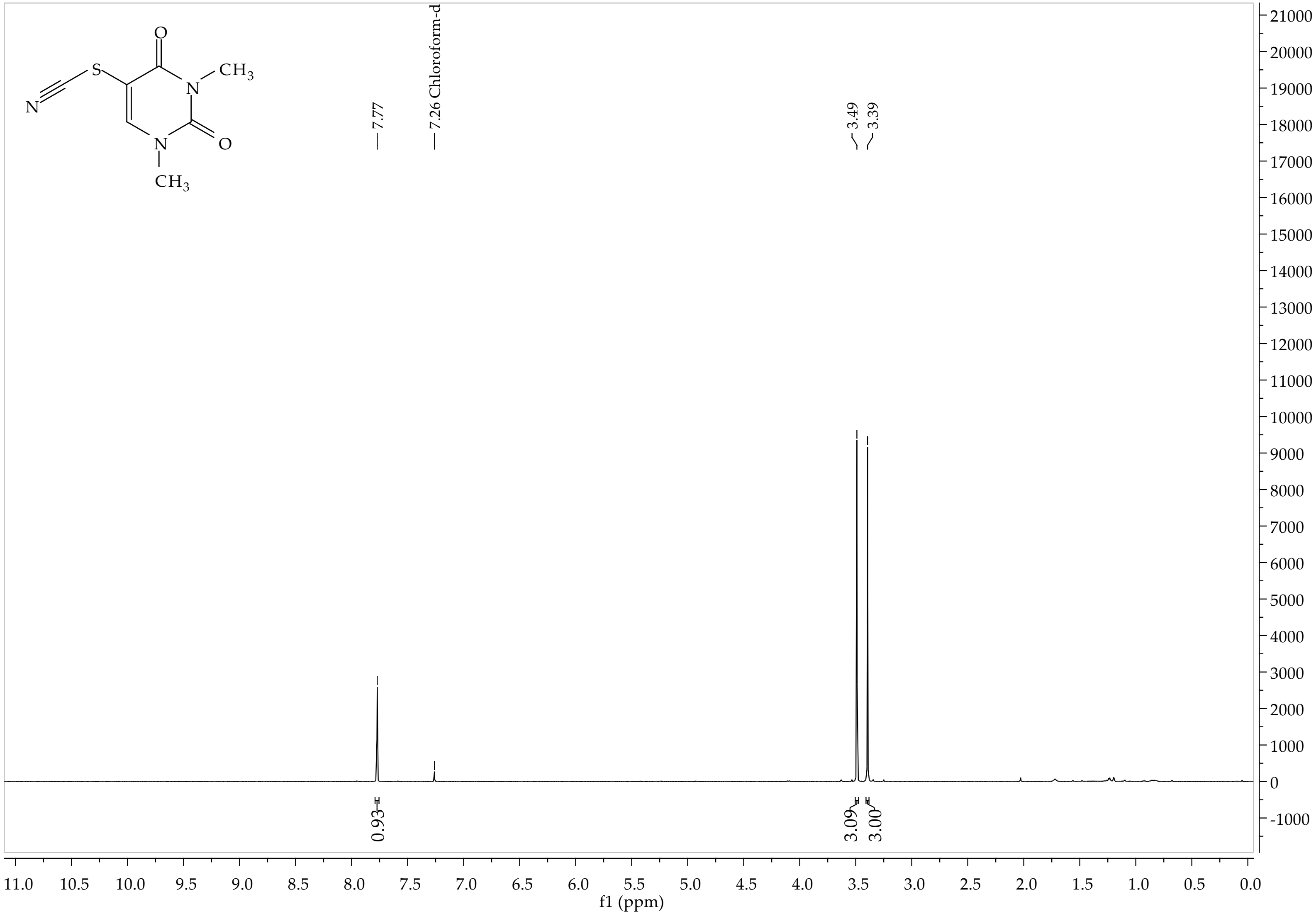

Figure S3. The 1H NMR spectrum of SCNdMeU sample. NMR spectra were measured in chloroform-d using 500 MHz spectrometer (Varian Unity Inova).

Table S1. Number of frames (out of 2703 total) containing 1, 2, 3, 4, 5 and 6 in a 10 Å water shell surrounding SCNdMeU.

**Number of water molecules**	**Number of frames**
1	2703
2	2702
3	2700
4	2636
5	2558
6	2184

Table S2. Clustering results of SCNdMeU$[H_2O]_n$, the cutoff used and number of clusters.

**Number of water molecules (n)**	**Cutoff**	**Clusters count (clusters having ≥10 structures count)**
1	0.08	12 (6)
2	0.12	18 (8)
3	0.15	18 (10)
4	0.21	18 (10)
5	0.27	16 (10)
6	0.31	17 (9)

Table S3. The AEA, VEA and VDE of SCNdMeU$[H_2O]_1$.

	**AEA (dG, eV)**	**VAE (dE, eV)**	**VDE (dE, eV)**
WAT1-1	-1.31	0.65	1.81
WAT1-2	-1.31	0.65	1.83
WAT1-3	-1.31	0.65	1.83
WAT1-4	-1.34	0.65	1.85
WAT1-5	-1.34	0.65	1.84
WAT1-6	-1.31	0.65	1.83
**Min. value**	-1.34	0.65	1.81
**Max. value**	-1.31	0.65	1.85
**Mean value**	-1.32	0.65	1.83
**Weighted avg.**	**-1.31**	**0.65**	**1.82**
**SD**	0.01	0.00	0.01

Table S4. The AEA, VEA and VDE of SCNdMeU$[H_2O]_2$.

	**AEA (dG, eV)**	**VAE (dE, eV)**	**VDE (dE, eV)**
WAT2-1	-1.47	0.64	2.05
WAT2-2	-1.47	0.64	2.05
WAT2-3	-1.47	0.64	2.07
WAT2-4	-1.53	0.68	2.07
WAT2-5	-1.40	0.35	2.07
WAT2-6	-1.35	0.44	2.06
WAT2-7	-1.51	0.64	2.09
WAT2-8	-1.38	0.48	2.02
**Min. value**	-1.53	0.35	2.02
**Max. value**	-1.35	0.68	2.09
**Mean value**	-1.45	0.57	2.06
**Weighted avg.**	**-1.47**	**0.63**	**2.06**
**SD**	0.06	0.11	0.02

Table S5. The AEA, VEA and VDE of SCNdMeU$[H_2O]_3$.

	**AEA (dG, eV)**	**VAE (dE, eV)**	**VDE (dE, eV)**
WAT3-1	-1.62	0.73	2.29
WAT3-2	-1.59	0.60	2.28
WAT3-3	-1.70	0.93	2.34
WAT3-4	-1.61	0.72	2.28
WAT3-5	-1.62	0.87	2.21
WAT3-6	-1.32	0.34	2.27
WAT3-7	-1.52	0.62	2.27
WAT3-8	-1.65	0.72	2.28
WAT3-9	-1.62	0.71	2.28
WAT3-10	-1.52	0.56	2.28
**Min. value**	-1.70	0.34	2.21
**Max. value**	-1.32	0.93	2.34
**Mean value**	-1.58	0.68	2.28
**Weighted avg.**	**-1.62**	**0.75**	**2.29**
**SD**	0.10	0.16	0.03

Table S6. The AEA, VEA and VDE of SCNdMeU$[H_2O]_4$.

	**AEA (dG, eV)**	**VAE (dE, eV)**	**VDE (dE, eV)**
WAT4-1	-1.75	1.15	2.49
WAT4-2	-1.59	0.53	2.55
WAT4-3	-1.81	0.96	2.56
WAT4-4	-1.81	0.96	2.56
WAT4-5	-1.82	0.97	2.54
WAT4-6	-1.70	0.77	2.39
WAT4-7	-1.71	0.58	2.53
WAT4-8	-1.54	0.40	2.53
WAT4-9	-1.66	0.57	2.52
WAT4-10	-1.70	0.54	2.45
**Min. value**	-1.82	0.40	2.39
**Max. value**	-1.54	1.15	2.56
**Mean value**	-1.71	0.74	2.51
**Weighted avg.**	**-1.74**	**0.92**	**2.53**
**SD**	0.09	0.24	0.05

Table S7. The AEA, VEA and VDE of SCNdMeU$[H_2O]_5$.

	AEA (dG, eV)	VAE (dE, eV)	VDE (dE, eV)
WAT5-1	-1.65	0.81	2.47
WAT5-2	-1.70	0.96	2.72
WAT5-3	-1.65	0.66	2.72
WAT5-4	-1.77	1.07	2.54
WAT5-5	-1.86	0.96	2.66
WAT5-6	-1.72	0.66	2.74
WAT5-7	-1.58	0.58	2.52
WAT5-8	-1.90	0.77	2.68
WAT5-9	-1.64	0.38	2.64
WAT5-10	-1.76	0.59	2.72
**Min. value**	-1.90	0.38	2.47
**Max. value**	-1.58	1.07	2.74
**Mean value**	-1.72	0.74	2.64
**Weighted avg.**	**-1.68**	**0.83**	**2.58**
**SD**	0.10	0.20	0.09

Table S8. The AEA, VEA and VDE of SCNdMeU$[H_2O]_6$.

	AEA (dG, eV)	VAE (dE, eV)	VDE (dE, eV)
WAT6-1	-1.52	0.50	2.65
WAT6-2	-1.80	0.82	2.60
WAT6-3	-1.94	1.28	2.83
WAT6-4	-1.86	1.11	2.69
WAT6-5	-1.74	0.52	2.80
WAT6-6	-1.97	0.99	2.79
WAT6-7	-1.75	0.61	2.78
WAT6-8	-1.69	0.63	2.82
WAT6-9	-1.38	0.41	3.00
**Min. value**	-1.97	0.41	2.60
**Max. value**	-1.38	1.28	3.00
**Mean value**	-1.74	0.76	2.77
**Weighted avg.**	**-1.66**	**0.69**	**2.68**
**SD**	0.18	0.28	0.11

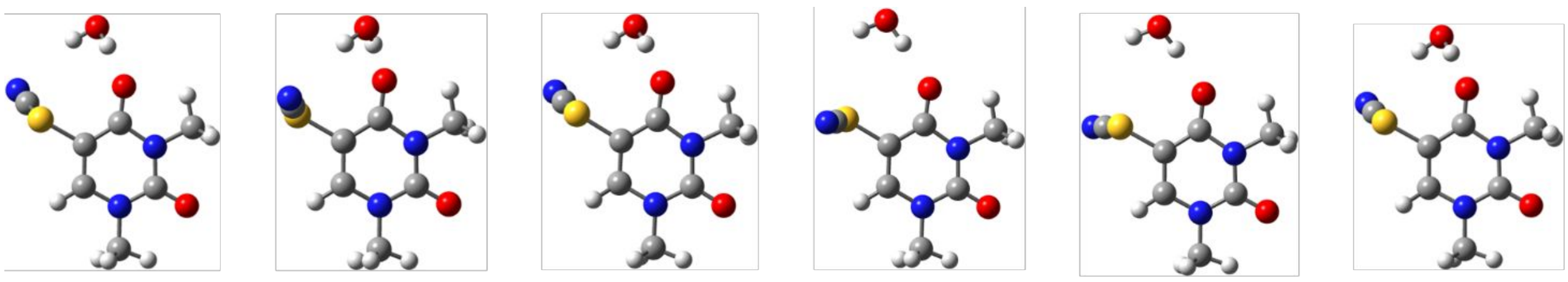

WAT1-1 73.4% | WAT1-2 13.4% | WAT1-3 9.5% | WAT1-4 1.9% | WAT1-5 1.2% | WAT1-6 0.6%

Figure S4. Visualization of SCNdMeU[$H_2O]_1^{\bullet-}$ structures hydrated with one water molecule, optimized at B3LYP/DGDZVP++ level, along with their names and relative cluster population.

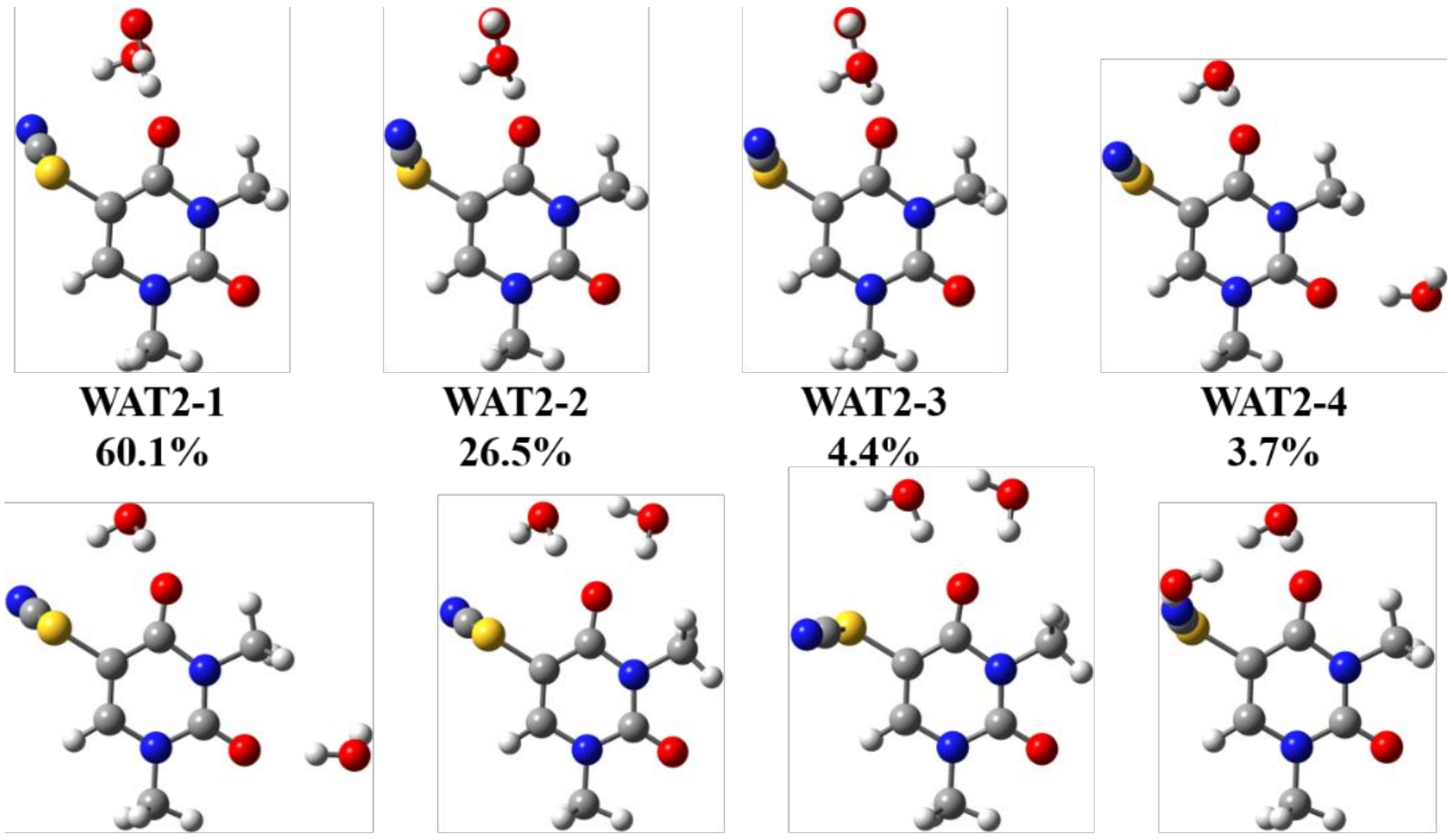

WAT2-1 60.1% | WAT2-2 26.5% | WAT2-3 4.4% | WAT2-4 3.7%

WAT2-5 3.7% | WAT2-6 0.7% | WAT2-7 0.5% | WAT2-8 0.4%

Figure S5. Visualization of SCNdMeU[$H_2O]_2^{\bullet-}$ structures hydrated with two water molecules, optimized at B3LYP/DGDZVP++ level, along with their names and relative cluster population.

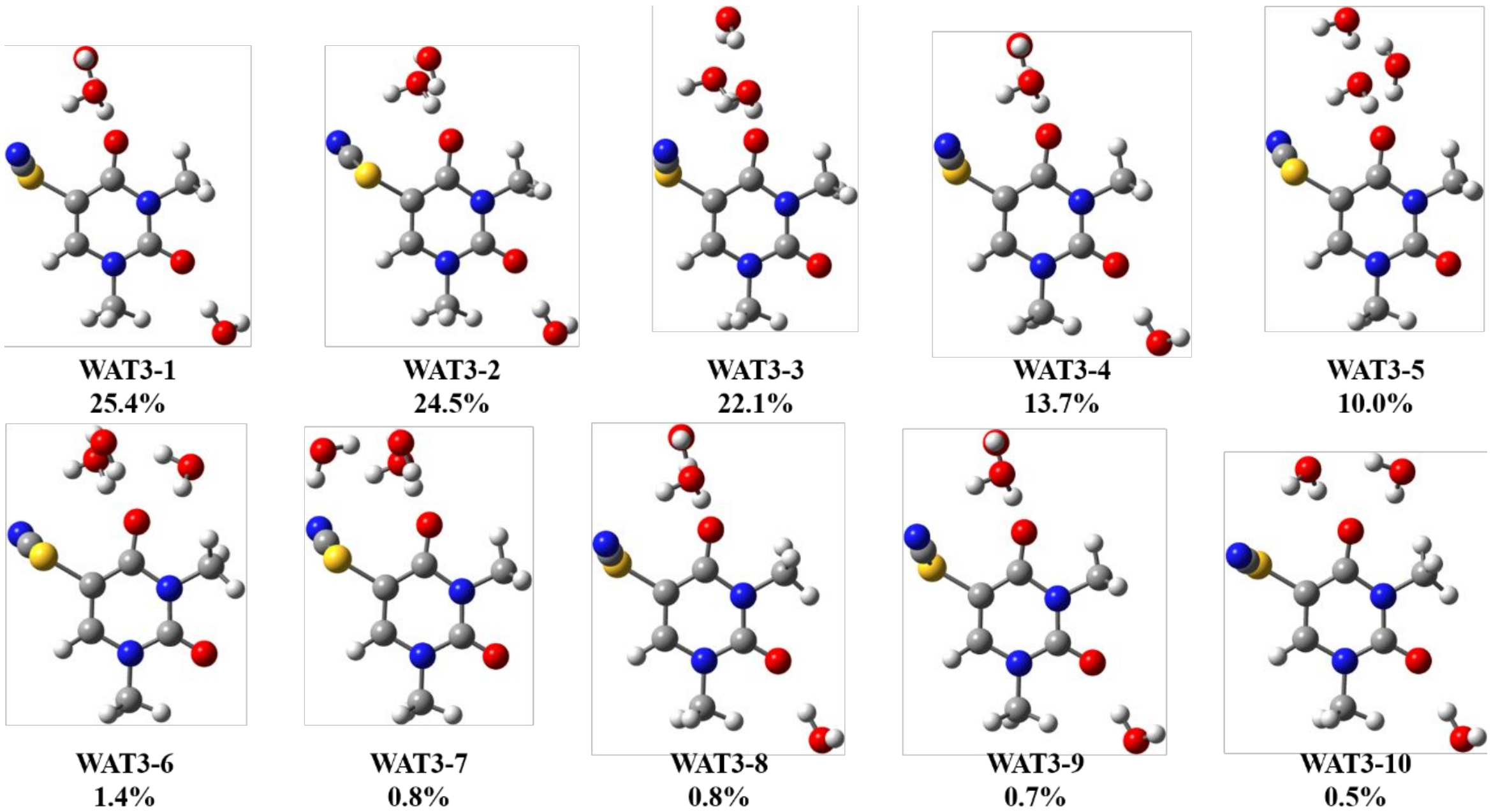

Figure S6. Visualization of SCNdMeU$[H_2O]_3^{\bullet-}$ structures hydrated with three water molecules, optimized at B3LYP/DGDZVP++ level, along with their names and relative cluster population.

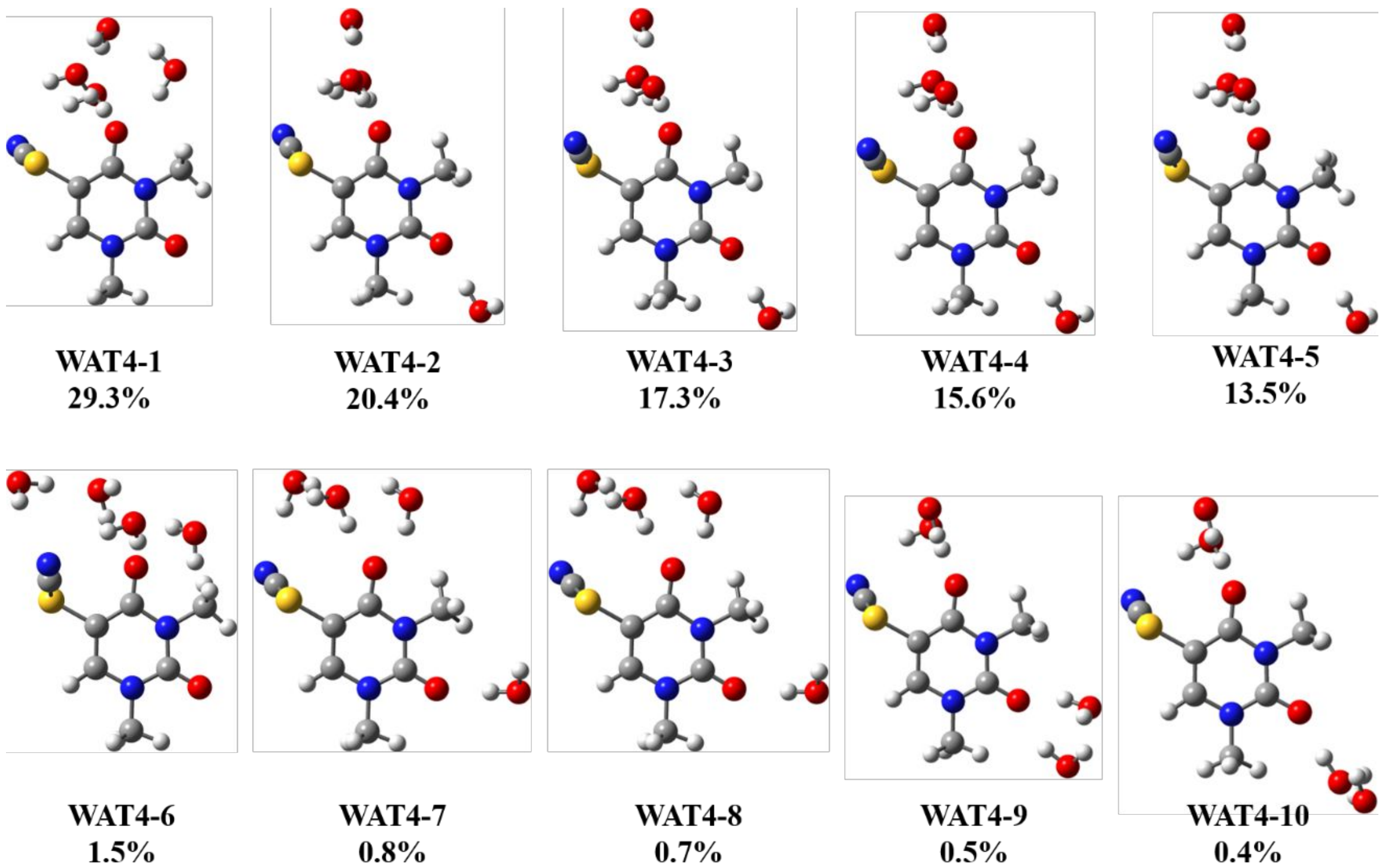

Figure S7. Visualization of SCNdMeU$[H_2O]_4^{\bullet-}$ structures hydrated with four water molecules, optimized at B3LYP/DGDZVP++ level, along with their names and relative cluster population.

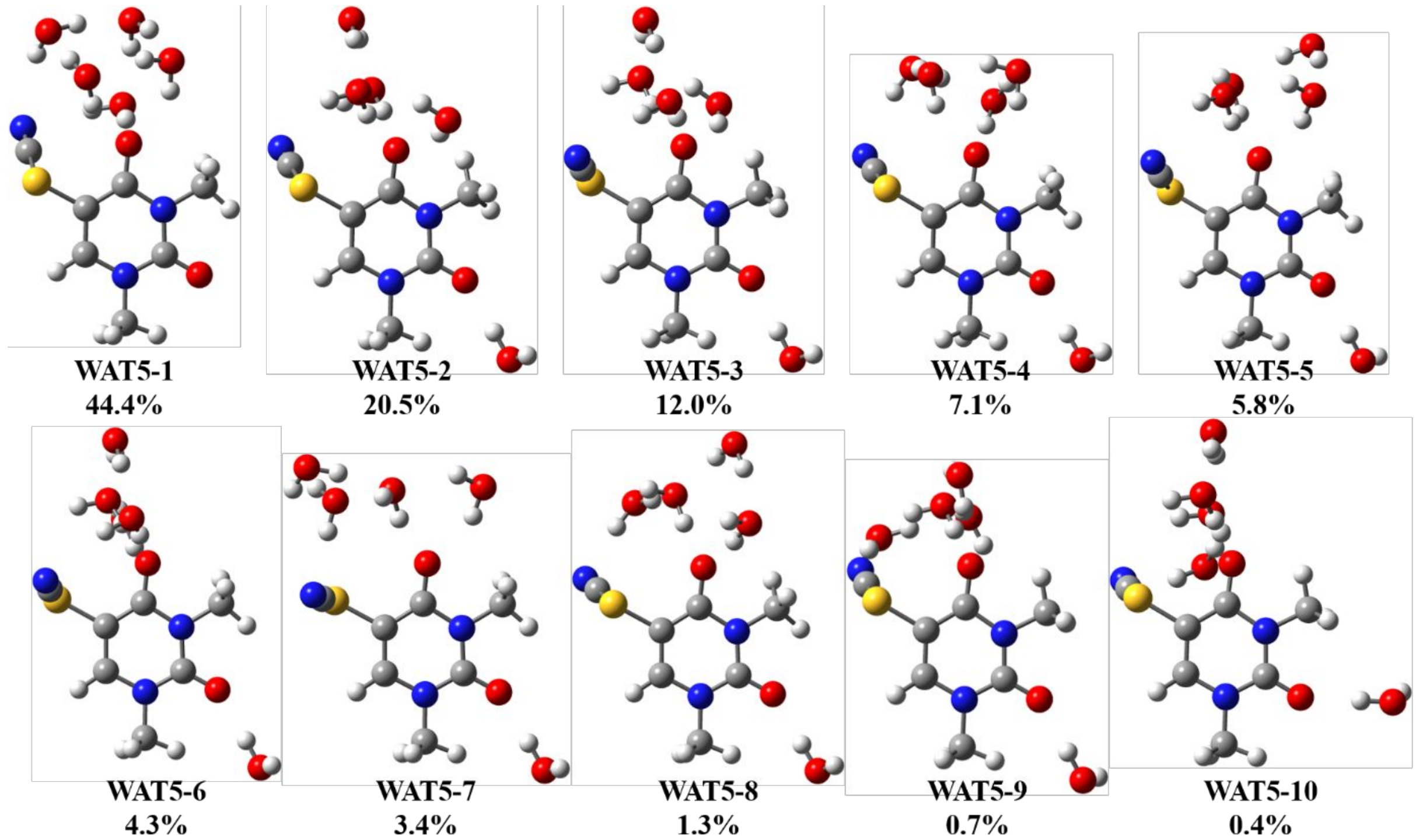

Figure S8. Visualization of SCNdMeU$[H_2O]_5^{\bullet-}$ structures hydrated with five water molecules, optimized at B3LYP/DGDZVP++ level, along with their names and relative cluster population.

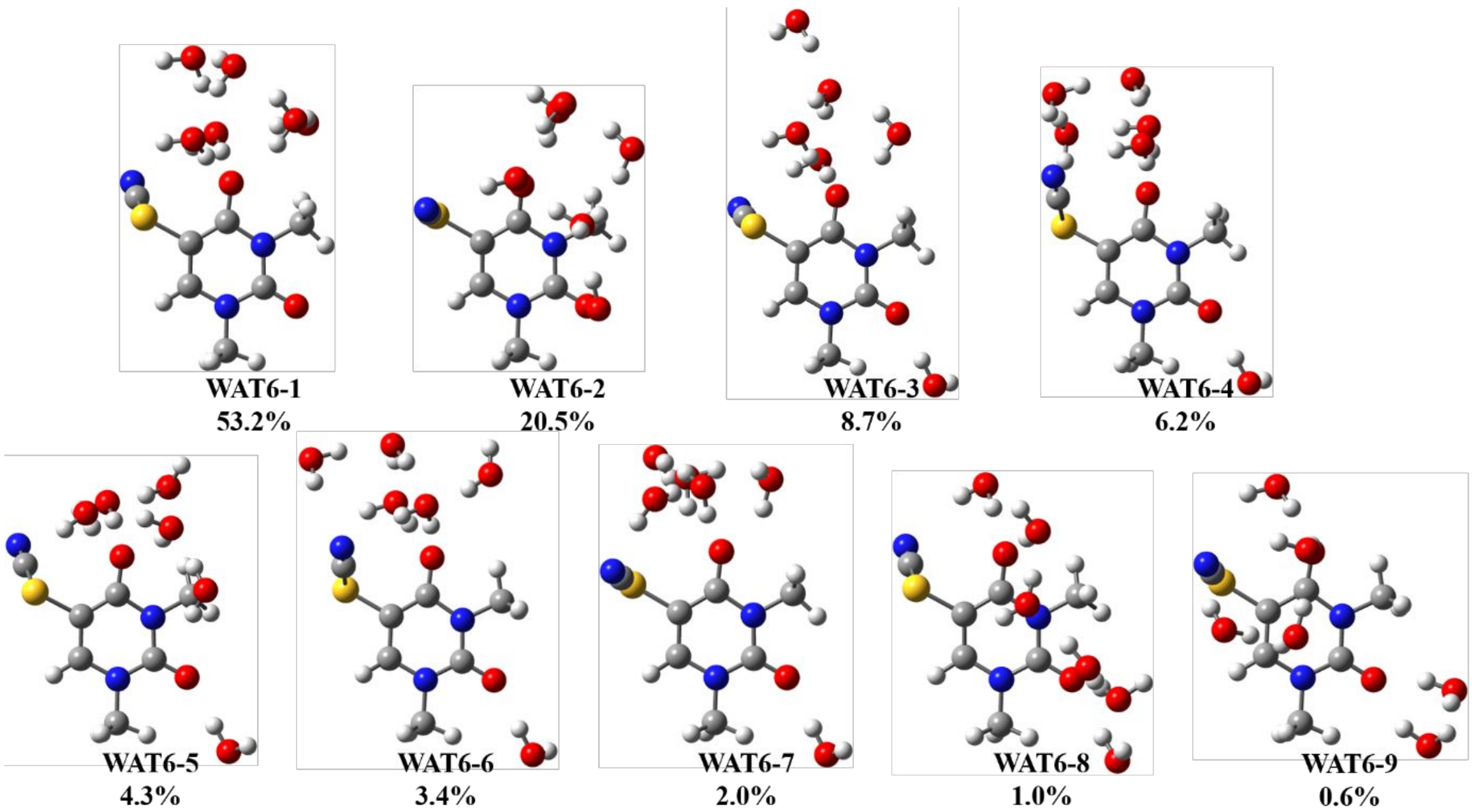

Figure S9. Visualization of SCNdMeU$[H_2O]_6^{\bullet-}$ structures hydrated with six water molecules, optimized at B3LYP/DGDZVP++ level, along with their names and relative cluster population.

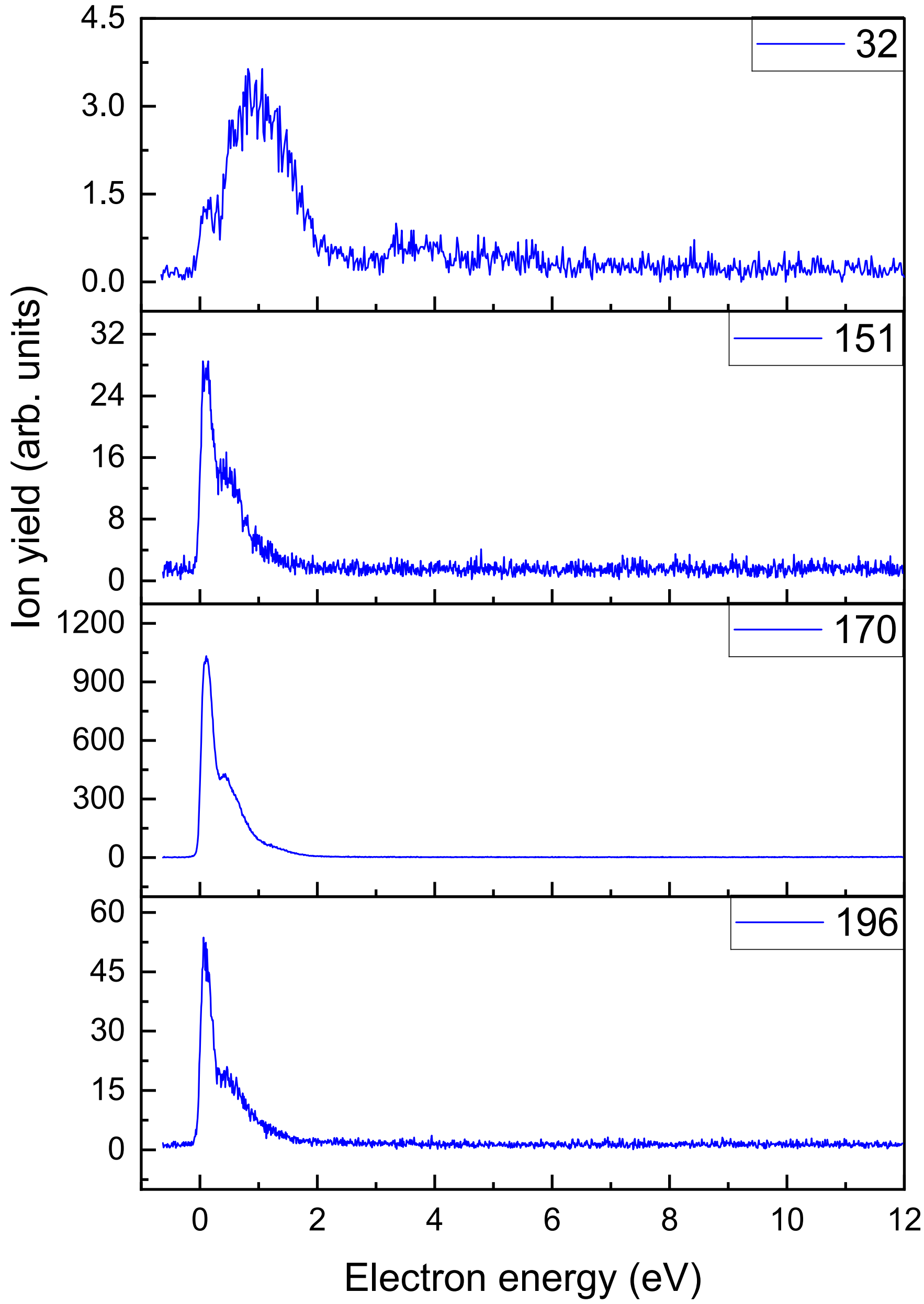

Figure S10. Anion efficiency curves of the fragment anions formed upon electron attachment to SCNdMeU: (m/z 32) $S^{\bullet-}$, (m/z 151) SCN^{-}, (m/z 170) SCNdMeUa-HCN$^{\bullet-}$. The anion efficiency curve for m/z 196 is also included (see comment S1).

Table S9. A summary of the observed fragment anions is provided, detailing their masses, structural assignments, and corresponding peak values on the anion efficiency curves, along with the experimental and calculated threshold energies (ΔE_0).

Mass (u)	Anion	Maxima of peak positions (eV)			Max inten. (Hz)	Threshold (eV)	
						Expt (351K)	Calcd (ΔE_0)
**32**	$[S]^{\bullet-}$	~0	1.0	3.7	3	~0.0	0.05
**151**	a[SCNdMeU-($NOCH_4$)a]$^-$	~0	0.4		29	~0.0	5.95
	b[SCNdMeU-($NOCH_4$)a]$^-$						5.08
	c[SCNdMeU-($NOCH_4$)a]$^-$						5.82
	d[SCNdMeU-($NOCH_4$)a]$^-$						5.86
	e[SCNdMeU-($NOCH_4$)a]$^-$						5.56
	f[SCNdMeU-($NOCH_4$)a]$^-$						3.95
	a[SCNdMeU-($NOCH_4$)b]$^-$						5.65
	b[SCNdMeU-($NOCH_4$)b]$^-$						4.78
	c[SCNdMeU-($NOCH_4$)b]$^-$						5.52
	d[SCNdMeU-($NOCH_4$)b]$^-$						5.56
	e[SCNdMeU-($NOCH_4$)b]$^-$						5.26
	f[SCNdMeU-($NOCH_4$)b]$^-$						3.65
	g[SCNdMeU-SCH_2]$^{\bullet-}$						-0.24
	h[SCNdMeU-SCH_2]$^{\bullet-}$						-0.33
**170**	[SCNdMeUa-HCN]$^{\bullet-}$	~0	0.4		1022	~0.0	-0.13
	[SCNdMeUb-HCN]$^{\bullet-}$						0.00
	[SCNdMeUc-HCN]$^{\bullet-}$						0.24
**196**	[SCNdMeUa-H]$^-$						1.43
	[SCNdMeUb-H]$^-$						2.38
	[SCNdMeUc-H]$^-$						2.64

COMMENT S1

The anion at m/z 32 (Figure S4) is assigned to the sulfur radical anion, $S^{\bullet-}$, formed upon cleavage of the C-S and S-CN bonds. The calculated threshold energy for this process is 0.05 eV (Table S1), indicating that this channel is accessible already at very low electron energies. The reaction may be written as eq S1:

$$e^- + \text{SCNdMeU} \rightarrow \text{SCNdMeU}^{\bullet-} \rightarrow [\text{SCNdMeU-S}] + S^{\bullet-} \qquad \textbf{(S1)}$$

The fragment ion at m/z 151 (Figure S4) originates from substantially more complex dissociation pathways. Several anionic structures were considered, corresponding either to loss of the $NOCH_4$ radical from conformers a or b or to elimination of the SCH_2 radical. Among the structures examined, the most favorable assignment is $[SCNdMeU\text{-}SCH_2]^{\bullet-}$, for which the calculated threshold is -0.33 eV (Table S1). This product therefore appears to arise from a multistep rearrangement accompanied by cleavage within the SCN substituent and demethylation at N1, as summarized in eq S2.

$$e^- + SCNdMeU \rightarrow SCNdMeU^{\bullet-} \rightarrow [SCNdMeU\text{-}SCH_2]^{\bullet-} + SCH_2 \qquad \textbf{(S2)}$$

Another experimentally observed product is the m/z 170 ion, assigned to $[SCNdMeU\text{-}HCN]^{\bullet-}$. Its formation involves concerted dissociation of the S-CN bond and a C-H bond, specifically through H abstraction from the N3 methyl group, followed by elimination of neutral HCN. The calculated threshold for this channel is slightly negative (-0.13 eV for the most favorable structure), which explains why this pathway remains accessible near 0 eV (eq S3).

$$e^- + SCNdMeU \rightarrow SCNdMeU^{\bullet-} \rightarrow [SCNdMeU\text{-}HCN]^{\bullet-} + HCN \qquad \textbf{(S3)}$$

The final minor anion signal considered here is observed at m/z 196, which would correspond to $[SCNdMeU\text{-}H]^-$ (eq S4) and formally associated with H-atom loss from C4. In this case, the calculated threshold is clearly positive (1.43 eV for the lowest-energy structure). The anion efficiency curve shows two overlapping features at ~0 eV and 0.4 eV. Since the calculated threshold is about 1 eV higher than the second peak position, we omit the assignment of both peaks to $[SCNdMeU\text{-}H]^-$.For the contribution near zero eV, partial overlap with the intense m/z 197 parent-anion signal due to the limited mass resolution may play a role, analogously to m/z 171 .

$$e^- + SCNdMeU \rightarrow SCNdMeU^{\bullet-} \rightarrow [SCNdMeU\text{-}H]^- + H^\bullet \qquad \textbf{(S4)}$$

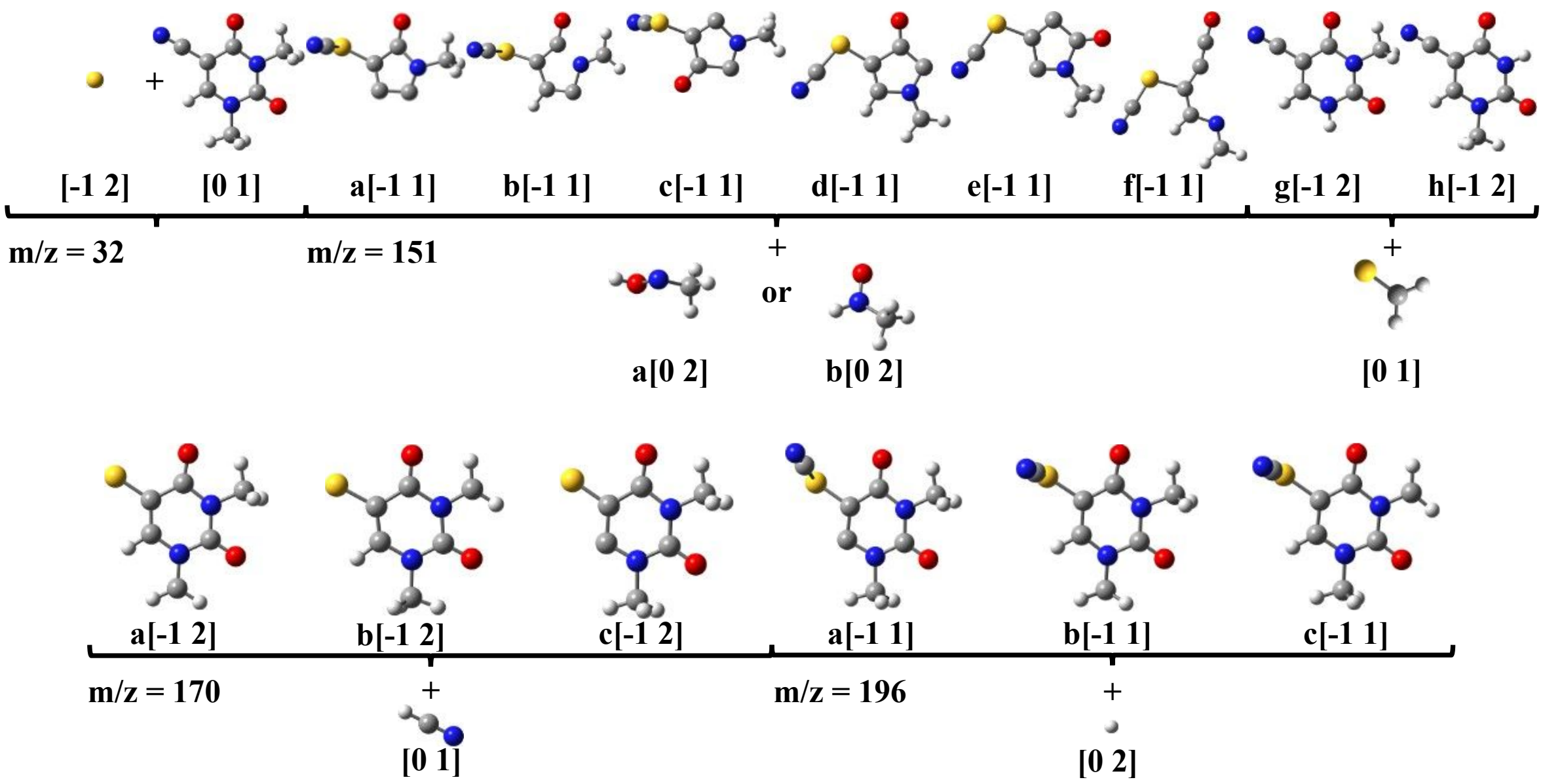

Figure S11. The experimentally observed fragments are identified by their m/z values: 32, 151 and 170. The detected anion signal at m/z 196 may not be ascribed to $[SCNdMeU\text{-}H]^-$, see comment S1. For each structure, charge and multiplicity are indicated in brackets. Atoms are color-coded as follows: hydrogen in white, carbon in gray, nitrogen in blue, and sulfur in yellow.

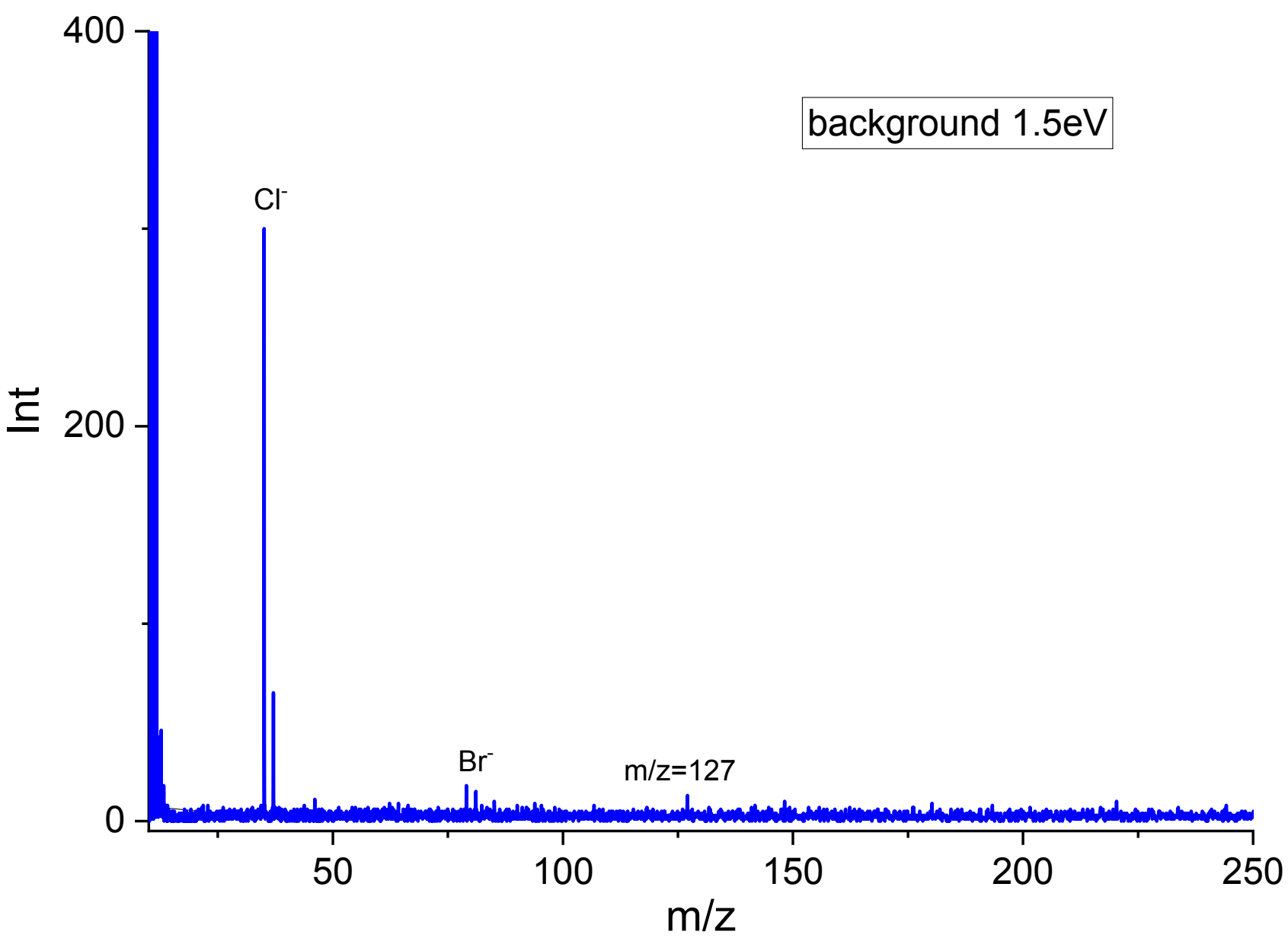

Figure S12. Negative ion MS at incident electron energy of 1.5 eV measured before hydration dependence spectra panels b),c),d) of the Figure 4 in main article.

Table S10. Thermodynamic and kinetic barriers of the DEA process (kcal/mol) for the two reaction pathways (S–CN^- and C–SCN^-), calculated at the B3LYP/DGDZVP++ level of theory for $SCNdMeU[H_2O]_1^{\bullet-}$. The highest and lowest ΔG* values are marked red and green, respectively.

	**CN^- dissociation channel**		**SCN^- dissociation channel**	
Structure	**ΔG* [kcal/mol]**	**ΔG [kcal/mol]**	**ΔG* [kcal/mol]**	**ΔG [kcal/mol]**
WAT1-1	2.29	-0.70	6.03	-0.15
WAT1-2	2.33	-0.66	5.52	0.55
WAT1-3	2.33	-0.66	5.52	0.55
WAT1-4	2.77	-1.22	5.06	1.13
WAT1-5	2.81	-1.19	5.80	-0.33
WAT1-6	2.33	-0.66	5.52	0.55
**Weighted avg.**	**2.31**	**-0.72**	**5.89**	**0.04**
**SD**	0.25	0.25	0.33	0.54

Table S11. Thermodynamic and kinetic barriers of the DEA process (kcal/mol) for the two reaction pathways (S–CN^- and C–SCN^-), calculated at the B3LYP/DGDZVP++ level of theory for $SCNdMeU[H_2O]_2^{\bullet-}$.The highest and lowest ΔG* values are marked red and green, respectively.

	**CN^- dissociation channel**		**SCN^- dissociation channel**	
Structure	**ΔG* [kcal/mol]**	**ΔG [kcal/mol]**	**ΔG* [kcal/mol]**	**ΔG [kcal/mol]**
WAT2-1	1.54	-2.38	6.07	-0.83
WAT2-2	1.54	-2.35	6.07	-0.82
WAT2-3	2.48	-1.20	6.08	-0.83
WAT2-4	3.67	1.01	6.87	1.66
WAT2-5	3.61	-2.49	6.88	1.66
WAT2-6	2.92	-2.44	6.44	0.70
WAT2-7	3.56	-0.07	5.69	0.55
WAT2-8	0.60	-4.66	4.25	-0.94
**Weighted avg.**	**1.76**	**-2.23**	**6.12**	**-0.62**
**SD**	1.16	1.62	0.84	1.14

Table S12. Thermodynamic and kinetic barriers of the DEA process (kcal/mol) for the two reaction pathways (S–CN$^-$ and C–SCN$^-$), calculated at the B3LYP/DGDZVP++ level of theory for SCNdMeU[H_2O]$_3^{\bullet-}$. The highest and lowest ΔG* values are marked red and green, respectively.

	**CN$^-$ dissociation channel**		**SCN$^-$ dissociation channel**	
Structure	**ΔG* [kcal/mol]**	**ΔG [kcal/mol]**	**ΔG* [kcal/mol]**	**ΔG [kcal/mol]**
WAT3-1	1.56	-2.04	6.50	-0.06
WAT3-2	1.40	-1.48	5.97	-0.27
WAT3-3	3.37	-1.02	6.11	1.46
WAT3-4	2.77	0.81	5.99	-0.26
WAT3-5	2.74	-0.94	5.61	0.67
WAT3-6	1.46	-3.73	5.80	0.45
WAT3-7	2.49	-2.07	6.18	1.27
WAT3-8	2.92	1.09	5.17	0.14
WAT3-9	1.42	-1.77	5.99	-0.26
WAT3-10	3.78	-0.43	7.15	1.62
**Weighted avg.**	**2.23**	**-1.18**	**6.10**	**0.30**
**SD**	0.88	1.35	0.52	0.71

Table S13. Thermodynamic and kinetic barriers of the DEA process (kcal/mol) for the two reaction pathways (S–CN$^-$ and C–SCN$^-$), calculated at the B3LYP/DGDZVP++ level of theory for SCNdMeU[H_2O]$_4^{\bullet-}$. The highest and lowest ΔG* values are marked red and green, respectively.

	**CN$^-$ dissociation channel**		**SCN$^-$ dissociation channel**	
Structure	**ΔG* [kcal/mol]**	**ΔG [kcal/mol]**	**ΔG* [kcal/mol]**	**ΔG [kcal/mol]**
WAT4-1	1.74	-3.18	5.49	-0.18
WAT4-2	3.74	0.58	6.83	1.91
WAT4-3	3.39	0.47	6.06	1.80
WAT4-4	3.28	0.55	6.06	1.80
WAT4-5	3.93	0.77	6.66	2.09
WAT4-6	2.39	-0.06	5.69	1.21
WAT4-7	3.75	-3.33	7.30	1.84
WAT4-8	3.85	-3.32	7.31	1.84
WAT4-9	3.88	2.21	7.17	0.51
WAT4-10	2.14	1.51	6.74	1.33
**Weighted avg.**	**3.02**	**-0.59**	**6.15**	**1.26**
**SD**	0.81	1.98	0.66	0.69

Table S14. Thermodynamic and kinetic barriers of the DEA process (kcal/mol) for the two reaction pathways (S–CN$^-$ and C–SCN$^-$), calculated at the B3LYP/DGDZVP++ level of theory for SCNdMeU$[H_2O]_5^{\bullet-}$. The highest and lowest ΔG* values are marked red and green, respectively.

	CN$^-$ dissociation channel		SCN$^-$ dissociation channel	
Structure	**ΔG* [kcal/mol]**	**ΔG [kcal/mol]**	**ΔG* [kcal/mol]**	**ΔG [kcal/mol]**
WAT5-1	1.71	-3.96	4.65	-1.64
WAT5-2	3.74	-0.77	7.42	1.74
WAT5-3	2.94	-0.35	7.85	2.15
WAT5-4	2.92	-0.65	5.30	0.53
WAT5-5	2.86	-0.36	6.22	0.33
WAT5-6	5.60	1.56	8.48	4.07
WAT5-7	1.26	-1.86	6.11	0.87
WAT5-8	3.84	-4.89	7.36	1.87
WAT5-9	2.94	-1.15	6.33	0.85
WAT5-10	3.75	-0.16	7.16	1.93
**Weighted avg.**	**2.62**	**-2.13**	**6.01**	**0.19**
**SD**	1.21	1.80	1.18	1.41

Table S15. Thermodynamic and kinetic barriers of the DEA process (kcal/mol) for the two reaction pathways (S–CN$^-$ and C–SCN$^-$), calculated at the B3LYP/DGDZVP++ level of theory for SCNdMeU$[H_2O]_6^{\bullet-}$. The highest and lowest ΔG* values are marked red and green, respectively.

	CN$^-$ dissociation channel		SCN$^-$ dissociation channel	
Structure	**ΔG* [kcal/mol]**	**ΔG [kcal/mol]**	**ΔG* [kcal/mol]**	**ΔG [kcal/mol]**
WAT6-1	4.46	-3.84	6.58	0.11
WAT6-2	4.71	0.19	5.68	2.13
WAT6-3	3.49	-2.44	6.03	-0.56
WAT6-4	2.50	-2.02	5.11	-0.62
WAT6-5	3.36	-2.25	11.71	4.19
WAT6-6	3.77	-1.03	6.17	1.40
WAT6-7	3.88	-8.39	7.52	2.13
WAT6-8	6.90	4.15	9.06	3.33
WAT6-9	0.95	-4.94	4.78	-1.35
**Weighted avg.**	**4.22**	**-2.67**	**6.50**	**0.71**
**SD**	1.62	3.26	2.20	1.81
